# Perfluoroalkyl and Polyfluoroalkyl Substances in the Environment: Terminology, Classification, and Origins

**DOI:** 10.1002/ieam.258

**Published:** 2011-07-25

**Authors:** Robert C Buck, James Franklin, Urs Berger, Jason M Conder, Ian T Cousins, Pim de Voogt, Allan Astrup Jensen, Kurunthachalam Kannan, Scott A Mabury, Stefan PJ van Leeuwen

**Affiliations:** † E.I. du Pont de Nemours & Co., Inc., DuPont Chemicals and FluoroproductsWilmington, Delaware, USA; ‡ CLF-Chem Consulting3 Clos du Châtaignier, BE-1390 Grez-Doiceau, Belgium; § Department of Applied Environmental Science (ITM), Stockholm UniversityStockholm, Sweden; ‖ ENVIRON InternationalIrvine, California, USA; # Institute for Biodiversity and Ecosystem Dynamics, University of AmsterdamAmsterdam, The Netherlands; †† Nordic Institute for Product Sustainability, Environmental Chemistry and Toxicology (NIPSECT)Frederiksberg, Denmark; ‡‡ Wadsworth Center, New York State Department of Health, and Department of Environmental Health Sciences, School of Public Health, State University of New York at AlbanyAlbany, New York, USA; §§ Department of Chemistry, University of TorontoToronto, Ontario, Canada; ‖‖ RIKILT—Institute of Food SafetyWageningen, The Netherlands

**Keywords:** Perfluoroalkyl, Polyfluoroalkyl, Terminology, Acronyms, PFAS

## Abstract

The primary aim of this article is to provide an overview of perfluoroalkyl and polyfluoroalkyl substances (PFASs) detected in the environment, wildlife, and humans, and recommend clear, specific, and descriptive terminology, names, and acronyms for PFASs. The overarching objective is to unify and harmonize communication on PFASs by offering terminology for use by the global scientific, regulatory, and industrial communities. A particular emphasis is placed on long-chain perfluoroalkyl acids, substances related to the long-chain perfluoroalkyl acids, and substances intended as alternatives to the use of the long-chain perfluoroalkyl acids or their precursors. First, we define PFASs, classify them into various families, and recommend a pragmatic set of common names and acronyms for both the families and their individual members. Terminology related to fluorinated polymers is an important aspect of our classification. Second, we provide a brief description of the 2 main production processes, electrochemical fluorination and telomerization, used for introducing perfluoroalkyl moieties into organic compounds, and we specify the types of byproducts (isomers and homologues) likely to arise in these processes. Third, we show how the principal families of PFASs are interrelated as industrial, environmental, or metabolic precursors or transformation products of one another. We pay particular attention to those PFASs that have the potential to be converted, by abiotic or biotic environmental processes or by human metabolism, into long-chain perfluoroalkyl carboxylic or sulfonic acids, which are currently the focus of regulatory action. The Supplemental Data lists 42 families and subfamilies of PFASs and 268 selected individual compounds, providing recommended names and acronyms, and structural formulas, as well as Chemical Abstracts Service registry numbers. Integr Environ Assess Manag 2011;7:513–541. © 2011 SETAC

## INTRODUCTION

“Fluorinated substances” is a general, nonspecific name that describes a universe of organic and inorganic substances that contain at least 1 F atom, with vastly different physical, chemical, and biological properties (Banks et al. 1994). Synonyms include “fluorochemicals” and “fluorinated chemicals.” A subset of fluorinated substances is the highly fluorinated aliphatic substances that contain 1 or more C atoms on which all the H substituents (present in the nonfluorinated analogues from which they are notionally derived) have been replaced by F atoms, in such a manner that they contain the perfluoroalkyl moiety C_n_F_2n+1_–. These compounds are hereafter referred to as “perfluoroalkyl and polyfluoroalkyl substances” and denoted by the acronym PFASs, justification for the choice of which is provided below. Since 1950, PFASs and surfactants and polymers made with the aid of PFASs have been widely used in numerous industrial and commercial applications (Kissa 2001). The C–F bond is extremely strong and stable (Smart 1994). The chemical and thermal stability of a perfluoroalkyl moiety, in addition to its hydrophobic and lipophobic nature, lead to highly useful and enduring properties in surfactants and polymers into which the perfluoroalkyl moiety is incorporated (Kissa 1994, 2001). Polymer applications include textile stain and soil repellents and grease-proof, food-contact paper (Rao and Baker 1994). Surfactant applications that take advantage of the unparalleled aqueous surface tension–lowering properties include processing aids for fluoropolymer manufacture, coatings, and aqueous film–forming foams (AFFFs) used to extinguish fires involving highly flammable liquids (Kissa 1994; Taylor 1999; Kissa 2001). Numerous additional applications have been described (3M Company 1999; Kissa 2001).

As a consequence of the widespread use of PFASs and their resulting emissions, a broad range of these substances have been detected in the environment, wildlife, and humans. The global extent of such contamination was first demonstrated for perfluorooctane sulfonic acid, C_8_F_17_SO_3_H (PFOS) in wildlife by Giesy and Kannan (2001). (It should be noted that, throughout this article, we refer to all PFASs containing an acid functionality as “acids,” regardless of whether or not they are likely to be highly or completely ionized in environmental or human matrices). At about the same time as the study by Giesy and Kannan, Hansen et al. (2001) discovered that PFOS, perfluorooctanoic acid (PFOA, C_7_F_15_COOH), and other PFASs were present in numerous samples of human blood purchased from biological supply companies. This latter study suggested that PFASs were responsible for a substantial fraction of the organic F detected in human serum in earlier pioneering studies on individuals not occupationally exposed to PFASs (e.g., Taves 1968; Belisle 1981). The blood of a group of fluorochemical industry workers had already been confirmed to contain PFOA (Ubel et al. 1980). The relative significance of various human exposure pathways for PFOS, PFOA, and related substances, i.e., via food, food-contact materials, drinking water, breast milk, airborne dust, air, and so forth, is a crucially important question that has been the focus of much research, reviewed recently by D'Hollander et al. (2010). Another important research topic, directly related to exposure of humans and wildlife, is the question of how and how fast PFOS and PFOA, as well as their homologues and precursors, are transported away from their emission sources over long distances in air and/or water (Armitage et al. 2006; Prevedouros et al. 2006; Wallington et al. 2006; Yarwood et al. 2007; Wania 2007; Schenker et al. 2008; Armitage et al. 2009a, 2009b; Stemmler and Lammel 2010).

The global regulatory community is specifically interested in “long-chain” perfluoroalkyl sulfonic acids (C_n_F_2n+1_SO_3_H, *n* ≥ 6, PFSAs) and perfluoroalkyl carboxylic acids (C_n_F_2n+1_COOH, *n* ≥ 7, PFCAs) and their corresponding anions (USEPA 2009; OECD 2011), which have been shown to be more bioaccumulative than their short-chain analogues (Martin et al. 2003a, 2003b; Conder et al. 2008; Olsen et al. 2009). PFOS and PFOA are the 2 “long-chain” perfluoroalkyl acids most often reported and discussed in the scientific literature.

As explained, for example, by Paul et al. (2009) and Prevedouros et al. (2006), the presence of PFOS, PFOA, and similar substances in the environment originates from the industrial use and environmental release of these substances, from use and disposal of consumer products that may contain them as an impurity, and from the abiotic or biotic degradation of larger functional derivatives and polymers that contain a perfluoroalkyl moiety and degrade in the environment to form PFOS, PFOA, and similar substances. These precursor substances are more commonly used commercially and may be released to the environment from industrial raw materials and products and from consumer products and articles.

Concerns about the potential environmental and toxicological impact of long-chain PFSAs and PFCAs have led to: 1) the phase-out of production of PFOS and related compounds and PFOA by their major global manufacturer in 2000 to 2002 (3M Company 2000a; USEPA 2000); 2) the conclusion of a stewardship agreement between the US Environmental Protection Agency (USEPA) and 8 leading global companies to reduce emissions and product content of PFOA and related chemicals by 95% by 2010 and to work toward their elimination by 2015 (USEPA 2006b); 3) a similar agreement between the Canadian environmental and health authorities and 5 companies to restrict PFCAs in products (Environment Canada 2010); 4) a European Union Marketing and Use Directive restricting the use of “perfluorooctane sulfonates” in the European Union (European Parliament 2006b); 5) the inclusion of PFOS in the Stockholm Convention on Persistent Organic Pollutants as an Annex B substance, i.e., restricted in its use (UNEP 2009); and 6) other regulatory and voluntary initiatives intended to reduce environmental emissions of this family of compounds.

The concern over potential environmental and human health impacts of PFASs has led to the launching of several large research programs to elucidate their environmental origin, fate, and impact, funded by various authorities in, for example, the European Union (de Voogt et al. 2006; de Voogt 2009), the United States (USEPA 2010), and Canada (INAC 2009). Moreover, alternative PFASs intended to be replacements for the long-chain PFSAs and PFCAs have been developed and implemented in certain cases (Visca et al. 2003; Higuchi et al. 2005; Hintzer et al. 2005; Brothers et al. 2008; Ishikawa et al. 2008; Peschka et al. 2008; Gordon 2011).

Since the first reports revealing the widespread global occurrence of PFOS in wildlife (Giesy and Kannan 2001) and the frequent detection of PFASs in human blood (Hansen et al. 2001) were published a decade ago, the scientific literature on the environmental and toxicological aspects of PFASs has burgeoned rapidly, and the rate of publication currently exceeds 400 articles per year. In the existing body of literature, including governmental reports, authors have created terminology, names, and acronyms to describe these substances. Unfortunately, inconsistencies have inevitably arisen between various groups of authors. In the absence of any concerted effort between scientists to agree on a common terminology to designate the substances, a given compound has often been denoted by a variety of different names and acronyms, or a given acronym has been used to represent different substances. In addition, names to describe broad groups of substances have proliferated that in some instances mistakenly portray substances that are very different from one another as being the same. As a result, the scientific literature for these substances has at times become confusing. There is a need for harmonized terminology, names, and acronyms that clearly and specifically describe PFASs.

## OBJECTIVES

The primary aim of this article is to recommend clear, specific, and descriptive terminology, names, and acronyms for PFASs, so as to promote a sound, unified understanding among all players in the PFAS industry, the environmental science related to it, and the bodies responsible for the regulation of chemicals, hence facilitating meaningful communication among all concerned.

A particular emphasis is placed on the long-chain perfluoroalkyl acids, substances related to the long-chain perfluoroalkyl acids, and substances intended as alternatives to the use of the long-chain perfluoroalkyl acids or their precursors. We trust that the terminology, names, and acronyms suggested will be broadly adopted by the “perfluoroalkyl and polyfluoroalkyl substances community” at large, leading to harmonized usage and the avoidance of misnomers. We have nevertheless refrained from creating an all new nomenclature but have retained—as far as possible—the most popular terms and acronyms used by authors to date. In other words, our proposals result from a pragmatic compromise among textbook and/or International Union for Pure and Applied Chemistry (IUPAC) chemical nomenclature, universal consistency, and frequently adopted “legacy” usage.

It is important to note that the substance terminology, names and acronyms proposed in this article are in no way intended to compete with or supplant IUPAC or Chemical Abstracts Service (CAS) nomenclature. The latter names are the designations of choice when a specific substance needs to be unequivocally identified, e.g., in official regulatory documents. Our intention is to provide terminology, names, and acronyms for pragmatic everyday use within the scientific community. Thus, for example, the IUPAC name for the substance C_8_F_17_SO_2_N(C_2_H_5_)CH_2_CH_2_OH is “*N*-ethyl-1,1,2,2,3,3,4,4,5,5,6,6,7,7,8,8,8-heptadecafluoro-*N*-(2-hydroxyethyl)octane-1-sulfonamide,” but it is more convenient to use the less rigorous but shorter designation “*N*-ethyl perfluorooctane sulfonamidoethanol” (or the corresponding acronym EtFOSE) for use in publications aimed at specialist readers. Rigor can always be ensured by appending the appropriate CAS Registry Number when each compound is first mentioned in a publication. We encourage this practice and provide CAS numbers for many commonly discussed compounds in the Supplemental Data.

In addition to recommending terminology, names, and acronyms, this article provides a brief review of certain topics useful for understanding the occurrence of and relationships between various families of PFASs in the environment. First, we describe the major commercial processes for synthesizing perfluoroalkyl moieties and the resulting compositions, including formation of isomers and/or homologues of the targeted main products. Second, we present the interrelationships between families of PFASs that may be precursors to or products of one another as a result of abiotic or biotic transformations that may occur under industrial, environmental, or metabolic conditions.

A large number of PFASs have been commercially produced (OECD 2007), and not all are covered here. We have included the main families, individual compounds, and their degradation products that have been detected in environmental and human samples related to long-chain perfluoroalkyl acids, precursors to these substances, and their short-chain fluorinated alternatives. We provide literature references for studies that demonstrate how one family of PFASs may be transformed into another under abiotic or biotic conditions, and/or report the presence of the various families in the environment or humans. Nevertheless, given the vast number of publications on the most common PFASs, such as the perfluoroalkyl sulfonic and carboxylic acids and their anions and salts, the reader is referred to published reviews and extensive surveys for comprehensive literature compilations for these compounds (e.g., Kannan et al. 2004; Houde et al. 2006; Lau et al. 2007; van Leeuwen and de Boer 2007; Jahnke and Berger 2009; Loos et al. 2009; Pistocchi and Loos 2009; Rayne and Forest 2009b; Butt, Berger, et al. 2010; de Voogt 2010; Kwok et al. 2010; Loos et al. 2010; Sturm and Ahrens 2010; Ahrens 2011; Houde et al. 2011). Furthermore, because an emphasis here is on how the various categories of PFASs are interrelated, our citations on transformation processes and environmental presence often refer to families of substances, so the reader should consult the original publications for details on individual substances.

It should be noted that in this article, the terms “substance,” “compound,” “chemical,” and “species” are used interchangeably for designating a given molecular structure, although it is recognized that in other contexts their meanings may not be identical. For example, in the European REACH legislation (European Parliament 2006a), a “substance” may include impurities and stabilizers in addition to the main constituent.

## KEY TERMINOLOGY AND USAGE ASSOCIATED WITH PERFLUOROALKYL AND POLYFLUOROALKYL SUBSTANCES

### Perfluoroalkyl and polyfluoroalkyl substances and perfluorocarbons defined

As defined above, PFASs are aliphatic substances containing one or more C atoms on which all the H substituents present in the nonfluorinated analogues from which they are notionally derived have been replaced by F atoms, in such a manner that PFASs contain the perfluoroalkyl moiety C_n_F_2n+1_–. More explicitly, we recommend that the family of compounds denoted by the acronym PFAS should encompass:

- Perfluoroalkyl substances, which are defined as aliphatic substances for which *all* of the H atoms attached to C atoms in the nonfluorinated substance from which they are notionally derived have been replaced by F atoms, except those H atoms whose substitution would modify the nature of any functional groups present. This usage is consistent with the definition of “perfluoro” and “perfluorinated” provided by Banks et al. (1994, p. 2).
- Polyfluoroalkyl substances, defined here as aliphatic substances for which all H atoms attached to at least one (but not all) C atoms have been replaced by F atoms, in such a manner that they contain the perfluoroalkyl moiety C_n_F_2n+1_– (e.g., C_8_F_17_CH_2_CH_2_OH). Thus, whereas the general chemical concept of “polyfluorination” embraces compounds containing “scattered” multiple F atoms (such as in CH_2_FCHFCHFCH_2_OH), as well as “grouped” ones (such as in CF_3_CF_2_CH_2_COOH), we consider that only those polyfluorinated substances having at least one perfluoroalkyl moiety C_n_F_2n+1_– belong to the PFAS family.

The differences between perfluoroalkyl and polyfluoroalkyl substances are illustrated by 2 concrete examples in Table 1.

**Table 1:** Examples of the correct and incorrect (or undesirable) uses of the proposed nomenclature for perfluoroalkyl and polyfluoroalkyl substances (PFASs)

	Example statements	
	
Example	Correct	Incorrect or *undesirable*
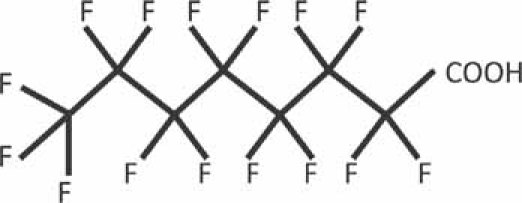	• Both are PFASs, within the family of perfluoroalkyl and polyfluoroalkyl substances	• Both are:
		– Perfluoroalkyl substances, chemicals, compounds
		– Perfluorinated substances, chemicals, compounds
		– Polyfluoroalkyl substances
		– Polyfluorinated substances
		– Fluorocarbons
		– Perfluorocarbons
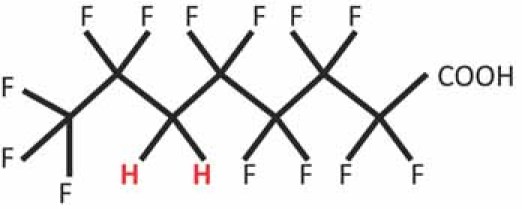	• Both are carboxylic acids	– *Fluorinated substances, chemicals, compounds*
		– Perfluorochemicals
		– Perfluorinated chemicals
		• Both contain fluorocarbons

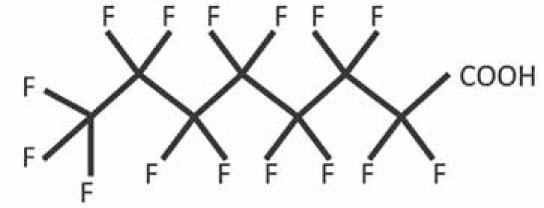	• All H atoms on all C atoms in the alkyl chain attached to the carboxylic acid functional group are replaced by F	• This is a:
		– *Perfluorinated substance, chemical, compound*
		– *Fluorinated substance, chemical, compound*
		– Fluorocarbon
	• This is a: PFAS, perfluoroalkyl acid (PFAA), perfluoroalkyl carboxylic acid (PFCA)	– Perfluorocarbon
	• Specifically, this is perfluorooctanoic acid, CAS number 335-67-1	

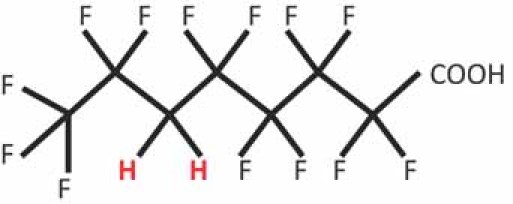	• The alkyl chain attached to the carboxylic acid functional group is polyfluorinated	• This is a:
		– *Polyfluorinated substance, chemical, compound*
		– *Fluorinated substance, chemical, compound*
	• This is a: PFAS, polyfluoroalkyl acid, polyfluoroalkyl carboxylic acid	– Perfluorinated substance, chemical, compound
		• *A portion of this compound is perfluorinated*
	• Specifically, this is 2,2,3,3,4,4,5,5,7,7,8,8,8- tridecafluorooctanoic acid	

Polyfluoroalkyl substances have the potential (i.e., the demonstrated or theoretical capability under appropriate conditions) to be transformed abiotically or biotically into perfluoroalkyl substances. For example, C_n_F_2n+1_SO_2_NHCH_2_CH_2_OH (a polyfluoroalkyl substance) may degrade in the environment to C_n_F_2n+1_SO_3_H (a perfluoroalkyl substance).

The general term “perfluoroalkyl(ated) substance,” with the acronym PFAS, was the first to be defined and widely used to describe the broad class of highly fluorinated substances observed in the environment (Hekster et al. 2002; Hekster et al. 2003). It has been employed by the groups of scientists who collaborated in the finalized European Union PERFORCE project (de Voogt et al. 2006) and others who have followed their example. Soon thereafter, many authors began using the acronym PFC and have defined it in many different ways. As a result, the meaning of the acronym PFC is unclear and not well defined. Moreover, we consider this choice to have been an unfortunate and inappropriate one, given that the acronym PFC has been used in official Kyoto Protocol documents since its adoption in 1997 to specifically designate perfluorocarbons (United Nations 1998), one of the families of greenhouse gases regulated by this important multilateral international agreement. Clearly, a given acronym may legitimately be used in different spheres of activity to denote different concepts, provided these activities are sufficiently disconnected from each other. However, both PFCs and PFASs belong to the overall family of fluorinated chemicals and, hence, are too closely related to share a common acronym. We, therefore, strongly urge the community to adopt henceforth the use of the term PFASs (singular PFAS) as an acronym for “perfluoroalkyl and polyfluoroalkyl substances” and the term PFCs (singular PFC) exclusively for “perfluorocarbons.” PFCs are notionally derived from hydrocarbons by replacing all H atoms by F atoms, so that they contain only the elements C and F, and functional groups are absent. Examples of PFCs are tetrafluoromethane (CF_4_), hexafluoroethane (C_2_F_6_), octafluorocyclobutane (*c*-C_4_F_8_), and perfluorodecalin (C_10_F_18_). Those PFCs that contain a C_n_F_2n+1_– moiety are, by definition, members of the PFAS family, but PFCs are chemically very stable substances, and it is uncertain whether any of them can actually degrade in the environment (e.g., in the upper atmosphere) to give functionalized PFASs such as PFCAs that might ultimately be deposited to the Earth's surface.

### “Fluorinated polymers” and “fluoropolymers” defined

We recommend using the broad generic term “fluorinated polymers” to encompass all polymers for which one or more of the monomer units contains the element F, in the backbone and/or in side chains. Fluorinated polymers may or may not be PFASs, depending on whether they contain perfluoroalkyl moieties.

In compliance with time-honored usage within the industry, we recommend further that the term “fluoropolymers” be applied only to a distinct subset of fluorinated polymers, namely, those made by (co)polymerization of olefinic monomers, at least one of which contains F bound to one or both of the olefinic C atoms, to form a carbon-only polymer backbone with F atoms directly attached to it, e.g., polytetrafluoroethylene.

### Chain length terminology

PFASs, especially the perfluoroalkyl acids and their anions, are frequently referred to as “long-chain” or “short-chain.” To avoid any subjectivity associated with these adjectives, we urge scientists to adopt the definition provided by the Organisation for Economic Co-operation and Development (OECD 2011), which stipulates that “long-chain” refers to:

- perfluoroalkyl carboxylic acids with eight carbons and greater (i.e., with 7 or more perfluorinated carbons) and,
- perfluoroalkane sulfonates with six carbons and greater (i.e., with 6 or more perfluorinated carbons).

The “long-chain” definitions for PFCAs and PFSAs are different in number of C atoms because a PFSA (e.g., PFHxS, C_6_F_13_SO_3_H) with a given number of carbons (6 in the example given) has a greater tendency to bioconcentrate and/or bioaccumulate than a PFCA with the same number of C atoms (e.g., PFHxA, C_5_F_11_COOH) (Martin et al. 2003a, 2003b). Although the OECD definition does not include perfluoroalkyl substances other than carboxylates and sulfonates, one may consider that a perfluoroalkyl chain with 7 or more C atoms, e.g., C_7_F_15_–, is, in any case, “long.”

### Linear and branched terminology

Many PFASs exist as families of isomers due to branching of the main C backbone (Alsmeyer et al. 1994). Linear isomers, for which there can only be 1 congener per C_n_ homologue group, are composed of carbons that are bonded to only 1 or 2 other C atoms. Branched isomers, for which there can be several or many congeners per C_n_ homologue group, are composed of C atoms that may be bound to more than 2 C atoms, resulting in a branching of the C backbone. For example, PFOS is routinely present in many environmental samples as a mixture of the linear isomer and 10 branched isomers (Riddell et al. 2009), whereas 89 congeners are theoretically possible (Rayne et al. 2008). To address the characterization of the numerous isomers and homologues arising during the electrochemical fluorination process (see below), a systematic numbering system for unequivocally identifying the linear and branched congeners of several families of PFASs has been proposed (Rayne et al. 2008). In the following text and in the Supplemental Data, we will designate perfluoroalkyl moieties, in general, by the formula C_n_F_2n+1_–, thereby including both linear and branched structures, even for substances that, given their manufacturing process (see discussion below), may be presumed to be predominantly linear, so that C_n_F_2n+1_– is equivalent to F(CF_2_)_n_–.

The mixture of linear and branched isomers presents challenges in providing an accurate quantification of many PFASs in environmental matrices (Riddell et al. 2009). Nevertheless, the study of linear and branched isomers is useful for understanding sources of PFASs (De Silva and Mabury 2004, 2006; De Silva et al. 2009; Benskin, De Silva, et al. 2010; Benskin, Yeung, et al. 2010), because the production of isomers varies by manufacturing process. The telomerization process produces primarily or exclusively linear PFASs, whereas the electrochemical fluorination process produces a mixture of branched and linear isomers, as discussed below.

### Use of acronyms for acids and their anions

Many PFASs are acids and may be present as protonated or anionic forms, or a mixture of both, depending on the pH of the environmental matrix and the compound's acid dissociation constant (p*K*_a_). The p*K*_a_ values for many of the PFASs (e.g., PFOA) are under review or are unknown, and for simplicity, we will refer to all PFASs with an acid functionality as “acids,” rather than as carboxylates, sulfonates, and so forth, although recognizing that the dissociated forms may well predominate in environmental and human matrices. Furthermore, given that these acids are generally analyzed as their anions (Larsen and Kaiser 2007), we recommend using the same substance acronym to cover both the protonated and ionized forms. However, an exception is made to this general rule when it is essential to make a distinction between the protonated acid form and the anionic form, such as when reporting physicochemical properties or modeling environmental fate and transport (Armitage et al. 2009b; Webster et al. 2010). In these cases, it is recommended to designate PFCA anions by removing the “A” from the individual substance acronym (e.g., PFO for perfluorooctanoate), maintain the original abbreviation for the acid (e.g., PFOA for perfluorooctanoic acid), and refer to both chemical forms using a collective abbreviation involving parentheses surrounding the “A,” e.g., PFO(A) for combined perfluorooctanoate and perfluorooctanoic acid. In the case of PFSAs, it is suggested to add the prefix “H-” to the generic substance acronym to form the abbreviation for the neutral species. This leads, for example, to the abbreviations H-PFOS, PFOS, and (H-)PFOS for the protonated, anionic, and combined forms of the 8-C PFSA, respectively.

### Surfactant terminology

Many PFASs are used as surfactants. Traditional surfactants comprise a water-soluble hydrophilic portion and a water-insoluble hydrophobic portion. Surfactants lower the surface tension of a liquid, or the interfacial tension between 2 liquids, or between a liquid and a solid. In fluorinated surfactants, the hydrophobic portion contains F bound to C, often as a perfluoroalkyl moiety. The extent of fluorination and location of the F atoms affect the surfactant properties. PFAS surfactants, often referred to as “fluorinated surfactants,” “fluorosurfactants,” “fluorinated tensides,” or “fluorotensides,” are superior in their aqueous surface tension reduction at very low concentrations and are useful as wetting and leveling agents, emulsifiers, foaming agents, or dispersants (Kissa 1994; Taylor 1999; Kissa 2001). The term “tenside” is encountered most frequently in publications of German origin, and the synonym “surfactant” is preferred in English. Examples of fluorinated surfactants are 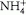 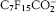 and Na^+^ 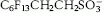.

### Terminology describing direct and indirect sources of PFASs to the environment

The sources of PFAS (e.g., PFOS or PFOA) emissions to the environment are from their purposeful manufacture, use, and disposal, from their being present as impurities in substances that are emitted to the environment or from precursor substances that degrade abiotically or biotically in the environment. Harmonizing the terminology for describing “sources” is needed. We recommend that the term “direct” emission sources should refer to emissions of a specific PFAS as such, throughout its product life cycle from manufacture to use and disposal, including emissions from a product in which the PFAS is present as an impurity. On the other hand, the term “indirect” emissions should apply to formation of a specific PFAS by transformation of precursor substances in the environment, wildlife, or humans, such as PFOA formed from the biotransformation of 8:2 fluorotelomer alcohol (FTOH), or C_4_F_9_COOH from the atmospheric degradation of perfluorobutane sulfonamidoethanol. These definitions depart somewhat from those of Prevedouros et al. (2006) who considered emissions of impurities present in a product to be “indirect.” These alternative definitions do not create large differences in the emissions allocated to direct and indirect sources in the case of PFOA, because the majority of direct emissions are derived from manufacturing sources.

## MANUFACTURING PROCESSES

For a better understanding of the environmental occurrence and behavior of PFASs, as well as the relationships between families of PFASs, it is useful to describe briefly the 2 principal manufacturing processes used to produce compounds containing perfluoroalkyl chains.

### Electrochemical fluorination

**Figure 1:**
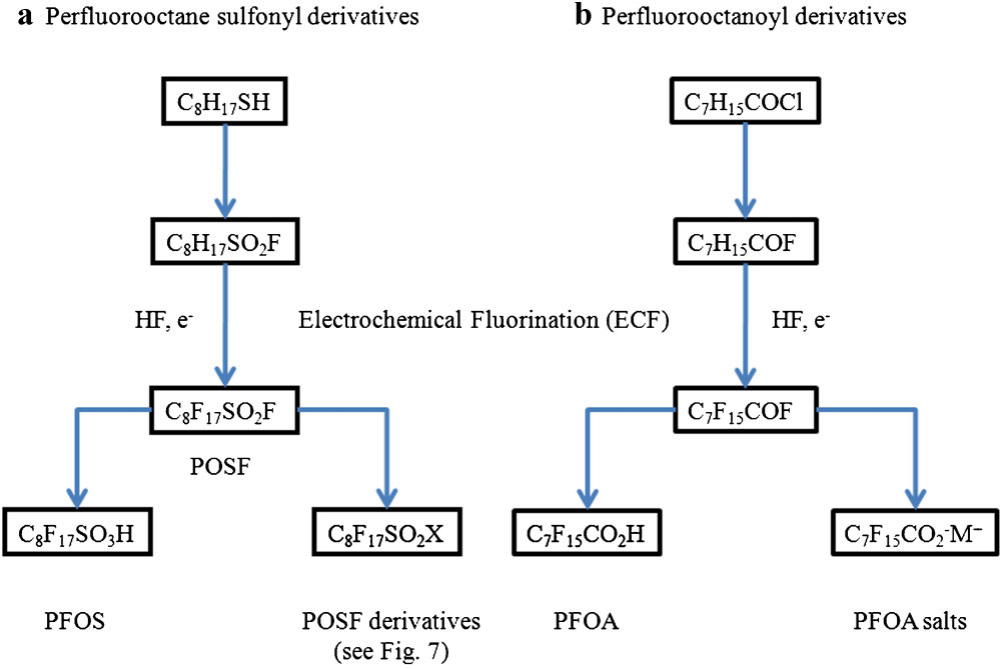
Synthesis, by electrochemical fluorination, of building blocks leading to PFOS, PFOA, and derivatives.

**Figure 2:**
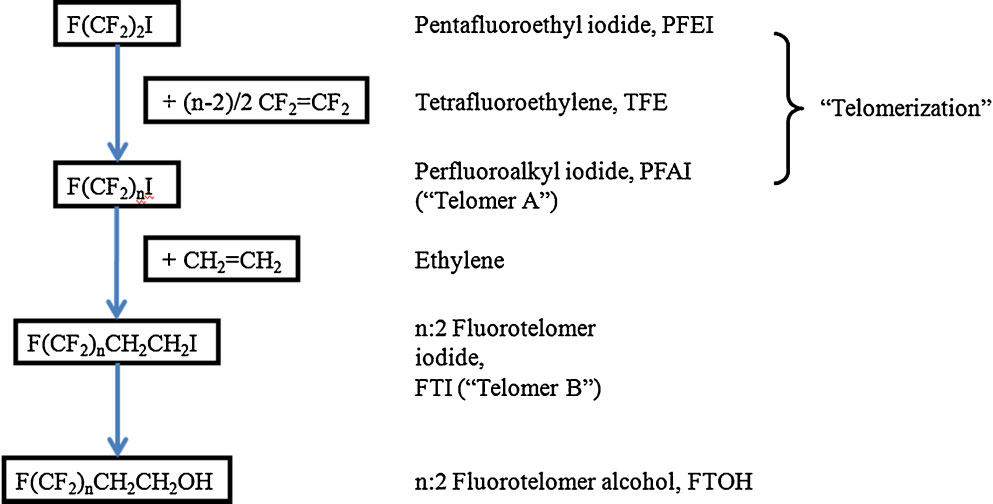
Synthesis, by telomerization, of building blocks leading to fluorotelomer alcohols.

Electrochemical fluorination (ECF) is a technology in which an organic raw material (e.g., octane sulfonyl fluoride [OSF], C_8_H_17_SO_2_F) undergoes electrolysis in anhydrous HF, leading to the replacement of all the H atoms by F atoms (Alsmeyer et al. 1994). The free-radical nature of the process leads to C chain rearrangement and breakage, resulting in a mixture of linear and branched perfluorinated isomers and homologues of the raw material, as well as PFCs and other species (Alsmeyer et al. 1994). The ratio of linear to branched perfluorinated C chains formed in the ECF process varies depending on how the process is controlled but is roughly 70% to 80% linear and 20% to 30% branched in the case of the synthesis of PFOS and PFOA (3M Company 1999; Reagen et al. 2007; Lehmler 2009; Benskin, De Silva, et al. 2010). The ECF of C_8_H_17_SO_2_F yields 1) perfluorooctane sulfonyl fluoride (POSF, C_8_F_17_SO_2_F), which is the major raw material used to manufacture PFOS (Figure 1a); 2) a series of functional raw materials such as sulfonamides, sulfonamido alcohols, and sulfonamido acrylate monomers; and 3) a family of surfactants and polymers derived therefrom (3M Company 1999; Lehmler 2005). Likewise, the ECF of octanoyl fluoride, C_7_H_15_COF, is the major historic process used to manufacture perfluorooctanoyl fluoride, C_7_F_15_COF, which is further reacted to make PFOA and its salts (Figure 1b) (Kissa 1994). The major global historic manufacturer using the ECF process produced 6-, 8-, and (to a lesser extent) 10-carbon perfluoroalkane sulfonyl derivatives and products therefrom (3M Company 2000c). In 2001, the company announced it would no longer manufacture these substances or PFOA. Others continued to use the ECF process to make these substances and there are now new manufacturers of both PFOS and PFOA. The major historic manufacturer is now making alternative products using the ECF process based on perfluorobutane, rather than perfluorooctane, sulfonyl chemistry (Renner 2006; Olsen et al. 2009; Ritter 2010).

### Telomerization

**Figure 3:**
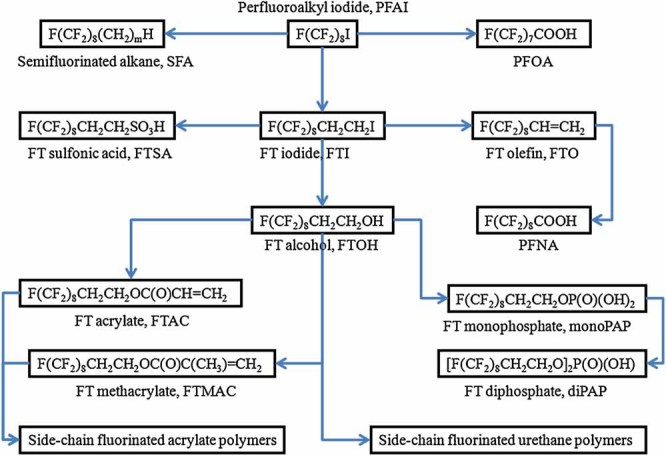
Perfluoroalkyl carboxylic acids and fluorotelomer (FT) derivatives synthesized from perfluoroalkyl iodides (PFAIs), exemplified for a starting PFAI with 8 C atoms. N.B. Names and acronyms for substance families are indicated. Those for the specific compounds shown can be found in the Supplemental Data.

Telomerization (Figure 2), which is a second important process for manufacturing perfluoroalkyl substances, is a technology in which a perfluoroalkyl iodide, C_m_F_2m+1_I (PFAI), most commonly pentafluoroethyl (or perfluoroethyl) iodide, C_2_F_5_I (PFEI), is reacted with tetrafluoroethylene, CF_2_=CF_2_ (TFE) to yield a mixture of perfluoroalkyl iodides with longer perfluorinated chains C_m_F_2m+1_(CF_2_CF_2_)_n_I. The starting iodide is referred to as the “telogen” and the TFE as the “taxogen.” The product perfluoroalkyl iodide mixture is often then reacted further, in a 2nd process step, where ethylene is inserted, to give C_m_F_2m+1_(CF_2_CF_2_)_n_CH_2_CH_2_I. The perfluoroalkyl iodides, C_m_F_2m+1_(CF_2_CF_2_)_n_I, commonly known as Telomer A, resulting from telomerization, the 1st step, and the “fluorotelomer iodides,” C_m_F_2m+1_(CF_2_CF_2_)_n_CH_2_CH_2_I, commonly known as Telomer B, formed in the 2nd step, are raw material intermediates used to produce additional building blocks that are further reacted to create a family of “fluorotelomer-based” surfactant and polymer products. This process is illustrated in Figure 2 for the synthesis of a fluorotelomer alcohol (FTOH), whereas Figure 3 shows how a range of products can be synthesized from the perfluoroalkyl iodide intermediate (exemplified for a starting PFAI with 8 C atoms).

It should be noted that, in the “*X*:*Y*” designation, e.g., 8:2 fluorotelomer alcohol (C_8_F_17_CH_2_CH_2_OH, 8:2 FTOH), used for naming fluorotelomer-based substances, *X* is the number of perfluorinated C atoms and *Y* is the number of nonfluorinated C atoms that originate from the commercial synthesis. As with products derived from ECF, the major global fluorotelomer manufacturers are making available alternative shorter-chain products, in this case based on 6 (rather than 8) perfluoroalkyl C atoms (Renner 2006; Ritter 2010).

The most widely used commercial telomerization process uses PFEI and TFE. When a linear telogen and taxogen are employed in the telomerization process, the resulting perfluoroalkyl iodides have exclusively linear perfluoroalkyl chains. If a branched and/or odd C number telogen, e.g., (CF_3_)_2_CFI, is employed and reacted with TFE, the resulting product mixture will be branched and/or will contain an odd number of C atoms, despite the incorporation of an even number of taxogen -CF_2_- units from the TFE. The extent to which branched and/or odd C number telogens may have been actually used in commercial practice is unclear. Such telogens have been described in patents (e.g., Katsushima et al. 1964; Millauer 1971; Grottenmüller et al. 2000), but this does not necessarily mean that they have been employed commercially. Nevertheless, in certain environmental samples, “isopropyl branched PFCA isomers,” i.e., ones with a terminal (CF_3_)_2_CF- group, have been observed, albeit at low levels compared to their linear counterparts, whereas other branched isomers were either absent or present at much lower levels. This is the case, inter alia, for PFCAs with 9, 11, or 13 C atoms, i.e., perfluorononanoic, perfluoroundecanoic, and perfluorotridecanoic acids (PFNA, PFUnDA, and PFTrDA, respectively), which are believed to be manufactured by the ozonation of a mixture of fluorotelomer olefins (FTOs, C_n_F_2n+1_CH=CH_2_) (Ukihashi et al. 1977; Aoyama and Chiba 1997) and which may be formed by the environmental transformation of telomer-derived precursor PFASs. The isopropyl branched isomers of these PFCAs observed in the environment (Furdui et al. 2008; De Silva et al. 2009; Benskin, De Silva, et al. 2010; Zushi et al. 2010) may therefore originate from the use of branched telogens for manufacturing specific isomers of PFNA, PFUnDA, and PFTrDA or their precursors. Nevertheless, the interpretation of branched-to-linear isomer concentration ratios is not straightforward, because certain environmental samples were found to contain up to 3 other PFNA isomers (for example) in addition to the linear and isopropyl branched forms (De Silva and Mabury 2006; Benskin et al. 2007; De Silva et al. 2009). Furthermore, the fact that individual isomers have different physicochemical properties means the patterns in the environment and biota will be transformed relative to the pattern in the emission source.

## FAMILIES OF PERFLUOROALKYL AND POLYFLUOROALKYL SUBSTANCES

**Figure 4:**
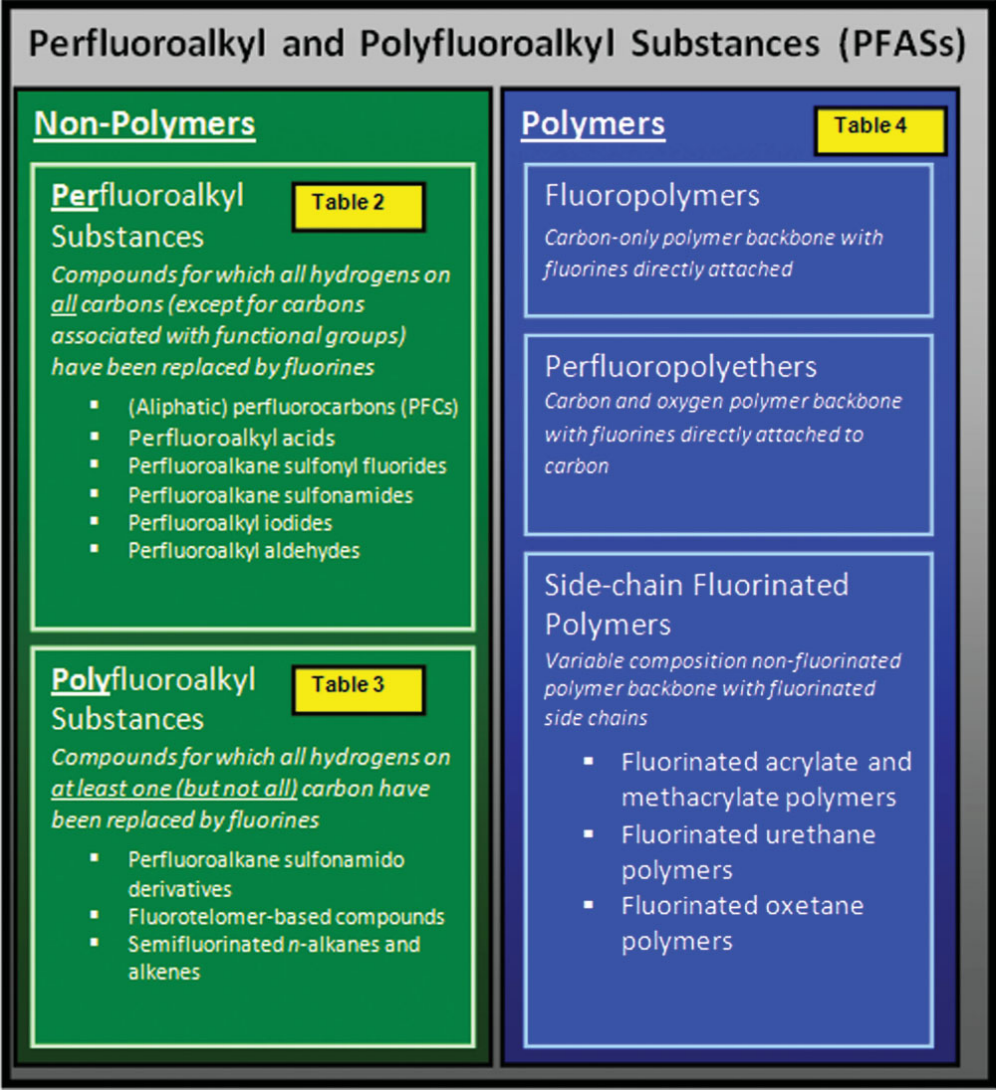
Classification hierarchy of environmentally relevant perfluoroalkyl and polyfluoroalkyl substances (PFASs).

There are numerous families of PFASs (Figure 4), each with many individual homologous members and isomers thereof (Tables 2, 3, and 4). This section provides a hierarchical overview of the common substance names, acronyms, and chemical formulas of those families of compounds and selected individual substances that have been detected in environmental and human matrices. The discussion includes references to manufacturing processes and uses for individual PFASs, as well as their environmental occurrence, for a better understanding of their environmental origin and how certain families and substances are related to one another. Another key point of the discussion is the likelihood that any or all members of PFAS groups have the ability to transform to the long-chain perfluorinated acids, provided, of course, that they have a long enough perfluoroalkyl moiety. A more comprehensive compilation of individual substances is given in the Supplemental Data, which also includes CAS registry numbers when assigned.

**Table 2:** Hierarchical overview of the nonpolymer perfluoroalkyl substances, compounds for which all H atoms on all C atoms in the alkyl chain attached to the functional group have been replaced with F

	Classification and chemical structure	C_n_F_2n+1_R, where R =	Examples	Uses
Perfluoroalkyl acids (PFAAs)	Perfluoroalkyl carboxylic acids (PFCAs)^a^	-COOH	Perfluorooctanoic acid (PFOA), C_7_F_15_COOH	Surfactant
		
	Perfluoroalkyl carboxylates (PFCAs)^a^	-COO^−^	Perfluorooctanoate (PFOA), C_7_F_15_COO^−^	
	
	Perfluoroalkane sulfonic acids (PFSAs)^b^	-SO_3_H	Perfluorooctane sulfonic acid (PFOS), C_8_F_17_SO_3_H	Surfactant
				
			Perfluorobutane sulfonic acid (PFBS), C_4_F_9_SO_3_H	
		
	Perfluoroalkane sulfonates (PFSAs)^b^	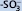	Perfluorooctane sulfonate (PFOS), 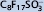	
				
			Perfluorobutane sulfonate (PFBS), 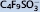	
	
	Perfluoroalkane sulfinic acids (PFSIAs)^b^	-SO_2_H	Perfluorooctane sulfinic acid (PFOSI), C_8_F_17_SO_2_H	Intermediate environmental transformation product
	
	Perfluoroalkyl phosphonic acids (PFPAs)^c^	-P(=O)(OH)_2_	Perfluorooctyl phosphonic acid (C8-PFPA) C_8_F_17_P(=O)(OH)_2_	Surfactant
	
	Perfluoroalkyl phosphinic acids (PFPIAs)^c^	-P(=O)(OH)(C_m_F_2m+1_)	Bis(perfluorooctyl) phosphinic acid (C8/C8-PFPIA) C_8_F_17_P(=O)(OH)(C_8_F_17_)	Surfactant

Perfluoroalkane sulfonyl fluorides (PASFs)^b^		-SO_2_F	Perfluorooctane sulfonyl fluoride (POSF), C_8_F_17_SO_2_F	Major raw material for surfactant and surface protection products
				
			Perfluorobutane sulfonyl fluoride (PBSF), C_4_F_9_SO_2_F	

Perfluoroalkane sulfonamides (FASAs)^b^		-SO_2_NH_2_	Perfluorooctane sulfonamide (FOSA), C_8_F_17_SO_2_NH_2_	Major raw material for surfactant and surface protection products

Perfluoroalkanoyl fluorides (PAFs)^b^		-COF	Perfluorooctanoyl fluoride (POF), C_7_F_15_COF	Major raw material for PFOA made by the ECF process; raw material for surfactant and surface protection products

Perfluoroalkyl iodides (PFAIs) (Telomer A)^c^		-I	Perfluorohexyl iodide (PFHxI), C_6_F_13_I	Major raw material for surfactant and surface protection products

Perfluoroalkyl aldehydes (PFALs) and aldehyde hydrates (PFAL·H_2_Os)^c^		-CHO and -CH(OH)_2_	Perfluorononanal (PFNAL), C_8_F_17_CHO	Intermediate environmental transformation product

a Substances originating by either electrochemical fluorination (ECF) or fluorotelomer processes;

b Substances originating by the ECF process;

c Substances originating by the fluorotelomer process.

**Table 3:** Hierarchical overview of the nonpolymer polyfluoroalkyl substances: compounds for which all H atoms on at least one (but not all) C atoms have been replaced with F

	Classification and chemical structure	C_n_F_2n+1_R, where R =	Examples	Uses
**Perfluoroalkane sulfonamido substances**^a^	*N*-Alkyl perfluoroalkane sulfonamides (MeFASAs, EtFASAs, BuFASAs)	-SO_2_NH(R′) where R′ = C_m_H_2m+1_ (*m* = 1,2,4)	*N*-Methyl perfluorooctane sulfonamide (MeFOSA), C_8_F_17_SO_2_N(CH_3_)H	Major raw material for surfactant and surface protection products
				
			*N*-Ethyl perfluorobutane sulfonamide (EtFBSA), C_4_F_9_SO_2_N(C_2_H_5_)H	
				
			*N*-Butyl perfluorooctane sulfonamide (BuFOSA), C_8_F_17_SO_2_N(C_4_H_9_)H	
	
	Perfluoroalkane sulfonamidoethanols (FASEs) and *N*-alkyl perfluoroalkane sulfonamidoethanols (MeFASEs, EtFASEs, BuFASEs)	-SO_2_N(R′)CH_2_CH_2_OH where R′ = C_m_H_2m+1_ (*m* = 0,1,2,4)	Perfluorooctane sulfonamidoethanol (FOSE), C_8_F_17_SO_2_NHCH_2_CH_2_OH	Major raw material for surfactant and surface protection products
				
			*N*-Ethyl perfluorobutane sulfonamidoethanol (EtFBSE), C_4_F_9_SO_2_N(C_2_H_5_)CH_2_CH_2_OH	
	
	*N*-Alkyl perfluoroalkane sulfonamidoethyl acrylates and methacrylates (MeFAS(M)ACs, EtFAS(M)ACs, BuFAS(M)ACs)	-SO_2_N(R′)CH_2_CH_2_O-C(O)CH = CH_2_ and -SO_2_N(R′)CH_2_CH_2_O-C(O)C(CH_3_) = CH_2_ where R′ = C_m_H_2m+1_ (*m* = 1,2,4)	*N*-Ethyl perfluorooctane sulfonamidoethyl acrylate (EtFOSAC), C_8_F_17_SO_2_N(C_2_H_5_)CH_2_CH_2_OC(O)CH = CH_2_	Major raw material for surfactant and surface protection products
	
	Perfluoroalkane sulfonamidoacetic acids (FASAAs) and *N*-alkyl perfluoroalkane sulfonamidoacetic acids (MeFASAAs, EtFASAAs, BuFASAAs)	-SO_2_N(R′)CH_2_COOH where R′ = C_m_H_2m+1_ (*m* = 0,1,2,4)	*N*-Ethyl perfluorooctane sulfonamidoacetic acid (EtFOSAA), C_8_F_17_SO_2_N(C_2_H_5_)CH_2_CO_2_H	Intermediate environmental transformation product

**Fluorotelomer substances**^b^	Semifluorinated *n*-alkanes (SFAs) and alkenes (SFAenes)	-(CH_2_)_m_H and –CH = CH(CH_2_)_m-2_H, with *m* = 2–16 and *n* = 6–16	Perfluorohexylhexadecane (F_6_H_16_), F(CF_2_)_6_(CH_2_)_16_H	Ski wax; medical applications

	n:2 Fluorotelomer iodides (n:2 FTIs) (Telomer B)	-CH_2_CH_2_I	8:2 Fluorotelomer iodide (8:2 FTI), C_8_F_17_CH_2_CH_2_I	Major raw material for surfactant and surface protection products

	n:2 Fluorotelomer olefins (n:2 FTOs)	-CH = CH_2_	6:2 Fluorotelomer olefin (6:2 FTO), C_6_F_13_CH = CH_2_	Raw material for surfactant and surface protection products

	n:2 Fluorotelomer alcohols (n:2 FTOHs)	-CH_2_CH_2_OH	10:2 Fluorotelomer alcohol (10:2 FTOH), C_10_F_21_CH_2_CH_2_OH	Major raw material for surfactant and surface protection products

	n:2 Unsaturated fluorotelomer alcohols (n:2 FTUOHs)	-CF = CHCH_2_OH	8:2 Unsaturated fluorotelomer alcohol (8:2 FTUOH), C_7_F_15_CF = CHCH_2_OH	Intermediate environmental transformation product

	n:2 Fluorotelomer acrylates (n:2 FTACs) and methacrylates (n:2 FTMACs)	-CH_2_CH_2_OC(O)CH = CH_2_ and -CH_2_CH_2_OC(O)C(CH_3_) = CH_2_	8:2 Fluorotelomer acrylate (8:2 FTAC), C_8_F_17_CH_2_CH_2_OC(O)CH = CH_2_	Major raw material for fluorotelomer-based polymers used in surface protection products
			6:2 Fluorotelomer methacrylate (6:2 FTMAC), C_6_F_13_CH_2_CH_2_OC(O)C(CH_3_) = CH_2_	

	n:2 Polyfluoroalkyl phosphoric acid esters, polyfluoroalkyl phosphates, fluorotelomer phosphates (PAPs)	(-CH_2_CH_2_O)_x_P(=O)(OH)_3-x_ where *x* = 1 or 2	8:2 Fluorotelomer phosphate monoester (8:2 monoPAP), C_8_F_17_CH_2_CH_2_OP(=O)(OH)_2_	Surfactant and surface protection products
			8:2 Fluorotelomer phosphate diester (8:2 diPAP), (C_8_F_17_CH_2_CH_2_O)_2_P(=O)OH	

	n:2 Fluorotelomer aldehydes (n:2 FTALs) and unsaturated aldehydes (n:2 FTUALs)	-CH_2_CHO and -CF = CHCHO	8:2 Fluorotelomer aldehyde (8:2 FTAL), C_8_F_17_CH_2_CHO	Intermediate environmental transformation product
			8:2 Fluorotelomer unsaturated aldehyde (8:2 FTUAL), C_7_F_15_CF = CHCHO	

	n:2 Fluorotelomer carboxylic acids (n:2 FTCAs) and unsaturated carboxylic acids (n:2 FTUCAs)	-CH_2_COOH and -CF = CHCOOH	8:2 Fluorotelomer carboxylic acid (8:2 FTCA), C_8_F_17_CH_2_COOH8:2 Fluorotelomer unsaturated carboxylic acid (8:2 FTUCA), C_7_F_15_CF = CHCOOH	Intermediate environmental transformation product

	n:3 Saturated acids (n:3 Acids) and n:3 Unsaturated acids (n:3 UAcids)	-CH_2_CH_2_COOH and -CH = CHCOOH	7:3 Acid, C_7_F_15_CH_2_CH_2_COOH	Intermediate environmental transformation product
			7:3 UAcid, C_7_F_15_CH = CHCOOH	

	n:2 Fluorotelomer sulfonic acids (n:2 FTSAs)	-CH_2_CH_2_SO_3_H	8:2 Fluorotelomer sulfonic acid (8:2 FTSA), C_8_F_17_CH_2_CH_2_SO_3_H	Surfactant and environmental transformation product

**Miscellaneous**	Polyfluoroalkyl ether carboxylic acids	For example: -O(C_m_F_2m_)OCHF(C_p_F_2p_)COOH	4,8-Dioxa-3*H*-perfluorononanoate, CF_3_OCF_2_CF_2_CF_2_OCHFCF_2_COOH	Alternative fluoropolymer processing aid (as ammonium salt)

a Substances originating by electrochemical fluorination (ECF) process;

b Substances originating by fluorotelomer process.

**Table 4:** Hierarchical overview of fluoropolymers, perfluoropolyethers, and side-chain–fluorinated polymers

		Example(s)	Uses
**Fluoropolymers:** Carbon-only polymer backbone with F directly attached to backbone C atoms		-(CF_2_CF_2_)_n_- Polytetrafluoroethylene (PTFE)	Plastics
		-(CH_2_CF_2_)_n_- Polyvinylidene fluoride (PVDF)	
		-(CH_2_CHF)_n_- Polyvinyl fluoride (PVF)	
		-(CF_2_CF_2_)_n_-(CF(CF_3_)CF_2_)_m_- Fluorinated ethylene propylene (FEP)	

**Perfluoropolyethers (PFPEs):** Ether polymer backbone with F atoms directly attached		Examples:	Functional fluids, surfactants, and surface protection products
		F-(C_m_F_2m_O-)_n_CF_3_	
		HOCH_2_O-[C_m_F_2m_O-]_n_CH_2_OH	
		-where C_m_F_2m_O represents -CF_2_O-, -CF_2_CF_2_O-, and/or -CF(CF_3_)CF_2_O- units distributed randomly along the polymer backbone	

**Side-chain–fluorinated polymers:** Nonfluorinated polymer backbone with fluorinated side chains, ending in -C_n_F_2n+1_	Fluorinated acrylate and methacrylate polymers	Acrylate:	Surfactants and surface protection products
		Backbone-CH-C(O)O-X-C_n_F_2n+1_	
		Methacrylate:	
		Backbone-C(CH_3_)-C(O)O-X-C_n_F_2n+1_	
		-where X is either -CH_2_CH_2_N(R′)SO_2_- with R′ = -C_n_H_2n+1_ (*n* = 0,1,2,4) or -CH_2_CH_2_-	
	
	Fluorinated urethane polymers	Backbone-NHC(O)O- X-C_n_F_2n+1_	Surfactants and surface protection products
		-where X is either -CH_2_CH_2_N(R′)SO_2_- with R′ = -C_n_H_2n+1_ (*n* = 0,1,2,4) or -CH_2_CH_2_-	
	
	Fluorinated oxetane polymers	Backbone-CH_2_OCH_2_-R	Surfactants and surface protection products
		-where R = -CF_3_, -C_2_F_5_ or -CH_2_C_4_F_9_	

First, we choose to make a fundamental distinction in substances by dividing them into 2 primary categories: nonpolymers and polymers (Figure 4). It is well accepted that polymers generally have very different physical, chemical, and biological properties than discrete chemical substances of low molecular weight (e.g., methyl methacrylate versus poly[methyl methacrylate]). There are various definitions of a polymer, but the basic concept describes a substance consisting of molecules characterized by the sequence of one or more types of monomer unit. Precise criteria for distinguishing polymers from nonpolymers have been established, for instance, under the European Union REACH legislation (ECHA 2008).

### Nonpolymer perfluoroalkyl and polyfluoroalkyl substances

#### Perfluoroalkyl acids

Perfluoroalkyl acids (PFAAs) occupy a prominent place in the literature on PFASs. The family of PFAAs includes perfluoroalkyl carboxylic, sulfonic, sulfinic, phosphonic, and phosphinic acids (Table 2). PFAAs are important both because they are highly persistent substances that have been directly emitted to the environment or are formed indirectly from the environmental degradation or metabolism of precursor substances, and because they (or their salts) are or have been used in a wide variety of industrial and consumer applications. Depending on their acid strength (p*K*_a_ value), PFAAs will dissociate to a greater or lesser extent to their anions in aqueous environmental media, soils, or sediments. The protonated and anionic forms have very different physicochemical properties. For instance, the perfluorooctanoate anion is highly water-soluble and has negligible vapor pressure, whereas perfluorooctanoic acid has very low water solubility and sufficient vapor pressure to partition out of water into air (Kaiser et al. 2005; Kaiser et al. 2006; Webster and Ellis 2010; Webster et al. 2010). However, for perfluoroalkyl carboxylic acids, there is an ongoing debate regarding what is the environmentally relevant p*K*_a_, with measured and estimated values varying by several log units for PFOA (Burns et al. 2008; Goss 2008; Cheng et al. 2009; Rayne and Forest 2010a).

##### Perfluoroalkyl carboxylic acids

Perfluoroalkyl carboxylic acids (PFCAs; Table 2), also known as perfluorocarboxylic acids or perfluoroalkanoic acids, have the general chemical formula C_n_F_2n+1_COOH. The most frequently discussed PFCA is PFOA, C_7_F_15_COOH. The ammonium salt of PFOA, ammonium perfluorooctanoate (APFO, 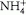 C_7_F_15_COO^−^) has been used for many decades as an essential “processing aid” in the manufacture of fluoropolymers such as polytetrafluoroethylene, by the dispersion (or emulsion) process (Kissa 1994; Fluoropolymer Manufacturing Group 2001). A chemically inert perfluorinated surfactant is chosen for this application to avoid reaction of the growing free-radical polymer chains with the processing aid, which would lead to a lowering of the molecular weight of the polymer produced. APFO and derivatives of it were also produced and marketed for fluorosurfactant use (3M Company 2000a). Between 1947 and 2002, APFO was manufactured by multiple companies around the world, probably mainly or exclusively by ECF of octanoyl fluoride. In 2002, the major global historic APFO manufacturer ceased its production (3M Company 2000a, 2000c). Thus, in addition to continued ECF-based APFO production from the remaining ECF producers, a process in which linear perfluorooctyl iodide (PFOI) synthesized by telomerization is converted into PFOA was brought on-stream in late 2002 to meet the need for this critical raw material (Prevedouros et al. 2006). This new telomerization-based process leads to only linear PFOA, whereas the ECF process produces a mixture of linear (70%–80%) and branched PFOA isomers.

Perfluorononanoic acid, C_8_F_17_COOH (PFNA) has also been manufactured and used (from 1975 onward) as its ammonium salt, 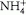 C_8_F_17_COO^−^ (APFN), principally for producing fluoropolymer dispersions, especially polyvinylidene fluoride (PVDF) (Prevedouros et al. 2006). It has also been marketed for general use as a fluorinated surfactant. A sample of commercial “APFN,” known as Surflon® S-111, has been analyzed and shown to contain significant proportions of the ammonium salts of longer PFCA homologues, especially those with 11 (PFUnDA) and 13 (PFTrDA) C atoms, which amounted to 20 and 5 weight percent of the mixture, respectively (Prevedouros et al. 2006; in the supporting information). The presence of these homologues with 2 and 4 additional C atoms, as confirmed by an industrial user (van der Putte et al. 2010), indicates that Surflon® S-111 is derived from a mixture of fluorotelomer-based precursors and, hence, suggests that it is constituted, predominantly or exclusively, of linear isomers. These conclusions are consistent with patents that claim manufacture of PFNA from telomer-based raw materials, namely by the oxidation of 8:2 fluorotelomer olefin, C_8_F_17_CH=CH_2_ (Ukihashi et al. 1977; Aoyama and Chiba 1997) or by the carboxylation of C_8_F_17_I (Nagasaki et al. 1988). The APFN commercial mixture has its own CAS Registry Number: 72968-38-8. Several publications report toxicological studies on the blend corresponding to this number, but do not provide information on the proportions or linearity of the homologues present (Mundt et al. 2007; Stump et al. 2008; Mertens et al. 2010).

In addition to their major commercial use as fluoropolymer processing aids and numerous industrial and consumer applications (Kissa 2001; Prevedouros et al. 2006), PFCAs are also the terminal degradation products from abiotic and biotic degradation of certain precursor PFASs. Such precursors include fluorotelomer alcohols (Hagen et al. 1981; Dinglasan et al. 2004; Ellis et al. 2004; Hurley et al. 2004; Wang et al. 2009; Liu et al. 2010), fluorotelomer acrylates (Butt et al. 2009; Butt et al. 2010b), fluorotelomer iodides (Young et al. 2008), fluorotelomer olefins (Nakayama et al. 2007), *N*-alkyl perfluoroalkane sulfonamides (Tomy, Tittlemier, et al. 2004; Martin et al. 2006; Plumlee et al. 2009), *N*-alkyl perfluoroalkane sulfonamidoethanols (D'eon et al. 2006; Plumlee et al. 2009), and polyfluoroalkyl phosphates (D'eon and Mabury 2007; Lee et al. 2010). Short-chain PFCAs (e.g., trifluoroacetic and pentafluoropropionic acids) may also be formed in the atmospheric degradation of certain hydrochlorofluorocarbons, hydrofluorocarbons, and fluorinated anesthetics (Boutonnet et al. 1999; Young and Mabury 2010) and perfluoro-2-methyl-3-pentanone (Jackson et al. 2011), as well as in the oxidative thermolysis of fluorinated polymers (Ellis et al. 2001). Yet, the quantitative attribution of sources of these short-chain PFCAs in the environment remains uncertain, and it is quite possible that further precursors will be identified. PFCA yields and rates of formation vary depending on the precursor substance and degradation conditions. Moreover, PFCAs and potential PFCA precursors, such as residual raw materials, may be present as impurities in commercial PFAS-based products (Washburn et al. 2005; Berger and Herzke 2006; Dinglasan-Panlilio and Mabury 2006; Larsen et al. 2006; Prevedouros et al. 2006; Schulze and Norin 2006; D'eon and Mabury 2007; Jensen et al. 2008; Fiedler et al. 2010). It was estimated that the majority (∼80%) of PFCAs have been released to the environment from fluoropolymer manufacture and use (Prevedouros et al. 2006). This percentage is, however, an overall value, heavily weighted toward the PFCAs with the greatest emissions, namely PFOA and (to a much lesser extent) PFNA. PFCAs with shorter or longer chain lengths are not known to arise primarily from fluoropolymer manufacture and use. Although in the same study (Prevedouros et al. 2006), indirect sources of PFOA and PFNA were estimated to be much less important than direct sources, there were larger uncertainties associated with the calculations for indirect sources and some recently identified precursors (e.g., polyfluoroalkyl phosphates) were excluded.

In 2006, 8 major global companies signed on to the USEPA “2010/2015 PFOA Stewardship Program” (USEPA 2006b) with commitments first to reduce emissions and product content of PFOA, higher homologues and precursors by 95% by 2010 and second to work toward the elimination of PFOA, higher homologues, and precursors by 2015. Companies have reported significant progress toward achieving these goals (Ritter 2010). Interestingly, coincident with these changes, there have been reports showing significantly increased levels of perfluorobutanoic acid (PFBA) in water (Möller et al. 2010) and air (Weinberg et al. 2011b) that are most likely associated with the conversion to shorter chain perfluoroalkyl products.

##### Perfluoroalkane (or -alkyl) sulfonic acids

Perfluoroalkyl sulfonic acids, C_n_F_2n+1_SO_3_H (PFSAs, Table 2), are the 2nd major PFAA family of significance. The alternative name perfluoroalkane sulfonic acid has been used most commonly in the literature, in line with IUPAC recommendations, and we will adopt it here. Perfluorooctane sulfonic acid C_8_F_17_SO_3_H (PFOS), is the PFSA that has commanded greatest attention beginning when it was first detected globally in biota (Giesy and Kannan 2001) and humans (Hansen et al. 2001). Subsequently, as stated above, the production of PFOS, perfluorohexane sulfonic acid (PFHxS), perfluorodecane sulfonic acid (PFDS), and the precursors of these PFSAs, was phased out by the major manufacturer in 2002 (3M Company 2000c; USEPA 2000). Nevertheless, PFOS and its derivatives are still manufactured in China (Han 2009), with a production of more than 200 tons of its precursor, perfluorooctane sulfonyl fluoride, in 2006 (Yue 2008). PFOS and related compounds have been the subject of a European Union directive restricting their production and use (European Parliament 2006b). Furthermore, PFOS has been classified as a persistent, bioaccumulative, and toxic substance (OECD 2002) and was recently added to Annex B (requiring use restrictions) of the Stockholm Convention list of persistent organic pollutants (UNEP 2009). Formerly, PFOS had a number of industrial and commercial applications (3M Company 1999; Kissa 2001; Brooke et al. 2004; Paul et al. 2009). However, the environmental and toxicological significance of PFOS, ubiquitous in the global environment, also results from its presence as an impurity in and formation from perfluorooctane sulfonamido precursor substances (3M Company 1999, 2000a; Lange 2000, 2001; Xu et al. 2004; Boulanger et al. 2005; D'eon et al. 2006; Rhoads et al. 2008; Xie et al. 2009) used in vastly greater quantities (Brooke et al. 2004; Paul et al. 2009). The global commercial production of PFOS and related compounds has, to our knowledge, been based essentially or perhaps exclusively on ECF. In this process, the electrolysis of a solution of octane sulfonyl fluoride in anhydrous HF leads to perfluorooctane sulfonyl fluoride, C_8_F_17_SO_2_F (POSF), the key intermediate from which all PFOS-related products are subsequently produced (3M Company 1999; Brooke et al. 2004; Lehmler 2005; Paul et al. 2009). The resulting PFOS, the precursor POSF and other derivatives manufactured from it, e.g., perfluorooctane sulfonamido derivatives such as amides, ethanol-substituted amides, and surfactant and polymeric products therefrom, may contain up to 30% branched isomers (Reagen et al. 2007), as well as additional C chain length homologues. For example, samples of the K salt of PFOS taken from the same 3M commercial lot were analyzed by 2 laboratories and found to have a purity of only 85% to 87% (representing the sum of all K-PFOS isomers), on the account of the presence mainly of C_2_-C_10_ PFSA homologues, but also of a range of PFCAs and other impurities (Seacat et al. 2003; Arsenault et al. 2008). Shorter perfluoroalkyl chain length products, notably perfluorobutane sulfonyl–based products, have been introduced as alternatives to the previously used compounds with 6 or more perfluorinated carbons, because these shorter chain length substances do not bioaccumulate due to their rapid elimination in multiple organisms tested (Olsen et al. 2009). This substitution is a consequence of the voluntary phase-out and/or subsequent regulatory restriction of PFOS-related substances and certain homologues with 5 to 7 and 9 or 10 perfluorinated C atoms (3M Company 2000b; Federal Register 2006b). Coincident with these changes, reports have shown significantly increased levels of perfluorobutane sulfonic acid (PFBS) in environmental waters, no doubt as a consequence of the conversion to 4-C ECF-derived perfluoro-butane sulfonyl products (Eschauzier et al. 2010; Möller et al. 2010).

##### Perfluoroalkane (or -alkyl) sulfinic acids

Perfluoroalkane sulfinic acids, C_n_F_2n+1_SO_2_H (PFSIAs; Table 2), are degradation products from commercial precursor compounds containing the C_n_F_2n+1_SO_2_N< moiety (e.g., perfluoroalkane sulfonamido ethanols, C_n_F_2n+1_SO_2_N(R)CH_2_CH_2_OH) (Lange 2000, 2001; Boulanger et al. 2005; Rhoads et al. 2008). PFSIAs have been detected in wastewater treatment plant (WWTP) effluents and in the environment (Ahrens et al. 2009b; Ahrens, Siebert, et al. 2009; Ahrens, Xie, et al. 2010).

##### Perfluoroalkyl phosphonic and phosphinic acids

Perfluoroalkyl phosphonic acids, O=P(OH)_2_C_n_F_2n+1_ (PFPAs; Table 2), and perfluoroalkyl phosphinic acids, O=P(OH)(C_n_F_2n+1_)(C_m_F_2m+1_) (PFPIAs; Table 2), are commercial surfactants manufactured and offered for a range of consumer and industrial uses (USEPA 2006a; Mason Chemical 2011). Blends of C_6_-C_12_ PFPAs and similar PFPIA blends, with CAS numbers 68412-68-0 and 68412-69-1, respectively, have been reported to have had annual production volumes in the range of tonnes to hundreds of tonnes in 1998 and 2002 (Howard and Muir 2010), but only recently have PFPAs been widely detected in environmental waters (D'eon et al. 2009b; D'eon and Mabury 2010) and PFPIAs in WWTP sludge (D'eon and Mabury 2010) and human serum (Lee and Mabury 2011).

#### Fluorotelomer-based products

The term “fluorotelomer-based products” describes a family of raw material building blocks, surfactant and polymeric products, and degradation products that all originate from the starting fluorotelomer raw material, perfluoroalkyl iodides (PFAIs), as depicted in Figures 2 and 3. As reviewed below, the degradation of fluorotelomer-based products is a potential source of PFCAs in the environment.

##### Perfluoroalkyl iodides, fluorotelomer iodides, and fluorotelomer olefins

Perfluoroalkyl iodides, C_n_F_2n+1_I (PFAIs; Table 2), and n:2 fluorotelomer iodides, C_n_F_2n+1_CH_2_CH_2_I (n:2 FTIs; Table 3), are the first 2 raw materials that lead to the family of polyfluoroalkyl “fluorotelomer-based” products. Both PFAIs and n:2 FTIs have recently been detected in air and soil near a fluorotelomer manufacturing facility in China (Ruan et al. 2010). Fluorotelomer olefins, C_n_F_2n+1_CH=CH_2_ (FTOs; Table 3) are synthesized by dehydrohalogenation of FTIs and may also be formed as an impurity in synthesizing fluorotelomer alcohols (FTOHs) from FTIs (Prevedouros et al. 2006). As stated above, processes for manufacturing PFNA by oxidation of 8:2 FTO have been patented and may have been used industrially. FTOs are hydrosilylated to create silanes that are used in a number of applications. FTOs have been detected in the atmosphere (Barber et al. 2007; Jahnke et al. 2007; Piekarz et al. 2007), where they degrade completely and rapidly, but are expected to form low yields of PFCAs (Young and Mabury 2010). The degradation scheme proceeds via a C_n_F_2n+1_CHO perfluoroalkyl aldehyde (PFAL; Table 2) intermediate (Vésine et al. 2000; Nakayama et al. 2007). The atmospheric transformation of FTIs probably is comparable to FTOs in the ultimate outcome, mineralization with low yield of PFCAs (typically 1%–10%), and involves both fluorotelomer aldehyde C_n_F_2n+1_CH_2_CHO (FTAL; Table 3) and PFAL intermediates, together with the fluorotelomer carboxylic acids, C_n_F_2n+1_CH_2_COOH (FTCAs; Table 3) (Young et al. 2008). FTIs may hydrolyze in natural waters (Rayne and Forest 2010c), and this transformation process would presumably lead to fluorotelomer alcohols and, hence, their degradation products, as discussed below.

##### Fluorotelomer alcohols and their acrylic, methacrylic, and phosphoric esters

The n:2 fluorotelomer alcohols, C_n_F_2n+1_CH_2_CH_2_OH (n:2 FTOHs; Table 3), are key raw materials in the production of n:2 fluorotelomer acrylates, C_n_F_2n+1_CH_2_CH_2_OC(O)CH=CH_2_ (n:2 FTACs) and n:2 fluorotelomer methacrylates, C_n_F_2n+1_CH_2_CH_2_OC(O)C(CH_3_)=CH_2_ (n:2 FTMACs) (Table 3 and Figure 3). The FT(M)AC monomers are copolymerized in an aqueous emulsion polymerization with a host of non-fluorinated acrylates and other monomers to manufacture fluorotelomer-based polymers (Rao and Baker 1994). These polymers provide water, oil, and stain repellency to textiles, leather, and paper substrates. There is extensive scientific literature on the environmental occurrence of FTOHs, particularly (but not exclusively) in air (Martin et al. 2002; Oono, Harada, et al. 2008; Oono, Matsubara, et al. 2008; Strynar and Lindstrom 2008; Jahnke et al. 2009; Mahmoud et al. 2009; Dreyer et al. 2010; Langer et al. 2010; Shoeib et al. 2010; Yoo et al. 2010; Ahrens et al. 2011; Haug et al. 2011; Shoeib et al. 2011; Yoo et al. 2011). Likewise, some FTACs (Piekarz et al. 2007; Oono, Harada, et al. 2008; Oono, Matsubara, et al. 2008; Dreyer, Weinberg, et al. 2009; Mahmoud et al. 2009; Dreyer et al. 2010; Langer et al. 2010; Weinberg et al. 2011a, 2011b) and FTMACs (Oono, Matsubara, et al. 2008) have also been detected in environmental samples. The chain lengths of these fluorotelomer derivatives may vary over a broad range. For example, FTOHs with up to 18 fluorinated C atoms have been reported as detected, but not quantified, in air from an occupational setting (Nilsson et al. 2010).

Fluorotelomer alcohol phosphate esters (Table 3) are commercial fluorinated surfactants that are made by many global suppliers by the same reactions employed for non-fluorinated phosphates and used primarily for their surface tension lowering, wetting, and leveling surfactant properties (Taylor 1999). The terminology we recommend for these substances is polyfluoroalkyl phosphoric acid monoesters (monoPAPs), (O)P(OH)_2_(OCH_2_CH_2_C_n_F_2n+1_), and diesters (diPAPs), (O)P(OH)(OCH_2_CH_2_C_n_F_2n+1_)(OCH_2_CH_2_C_m_F_2m+1_). They may also be called n:2 fluorotelomer monophosphates and diphosphates. These compounds have been used as grease-proofing agents for food-contact paper (D'eon and Mabury 2007; Begley et al. 2008; FDA 2009; Lee et al. 2010; Lee and Mabury 2011), often as blends of varying perfluoroalkyl chain length and as salts (e.g., of diethanolamine). One specific use of monoPAPs and diPAPs that has led to their widespread presence in the environment is as an approved defoaming adjuvant in pesticide formulations. Approval for this use has now been rescinded (Federal Register 2006a). Recently, diPAPs have been reported detected in human serum at concentrations in some cases comparable to those of PFOA and in WWTP sludge at much greater levels than PFOA (D'eon et al. 2009a; Lee and Mabury 2011).

##### Semifluorinated alkanes and alkenes

Diblock semifluorinated *n*-alkanes (SFAs), F(CF_2_)_n_(CH_2_)_m_H (or, briefly, F_n_H_m_; Table 3), are a class of chemicals that are manufactured with a wide variety of chain lengths, depending on the intended use, by adding an olefin to a perfluoroalkyl iodide followed by reductive dehalogenation (Napoli 1996). These reactions also lead to semifluorinated *n*-alkenes (SFAenes), F(CF_2_)_n_CH=CH(CH_2_)_m-2_H (or, briefly, F_n_H_m_ene), as byproducts (Coe and Milner 1972). Since the 1990 s, industrial mixtures of long-chain SFAs (≥22 C atoms) have been applied in ski waxes, because they reduce friction and repel dirt due to their extremely low surface tension (Rogowski et al. 2007). Shorter-chain SFAs are used in medicinal applications (e.g., Kirchhof et al. 2002). In fluorinated ski waxes, up to 15% of SFAs are mixed with normal paraffins. The presence of SFAs in snow and soil samples from a ski area in Sweden has recently been demonstrated (Plassmann and Berger 2010).

##### Degradation products of fluorotelomer alcohols and their esters: Fluorotelomer aldehydes and acids, perfluoroalkyl aldehydes, perfluoroalkyl carboxylic acids, and so forth

**Figure 5:**
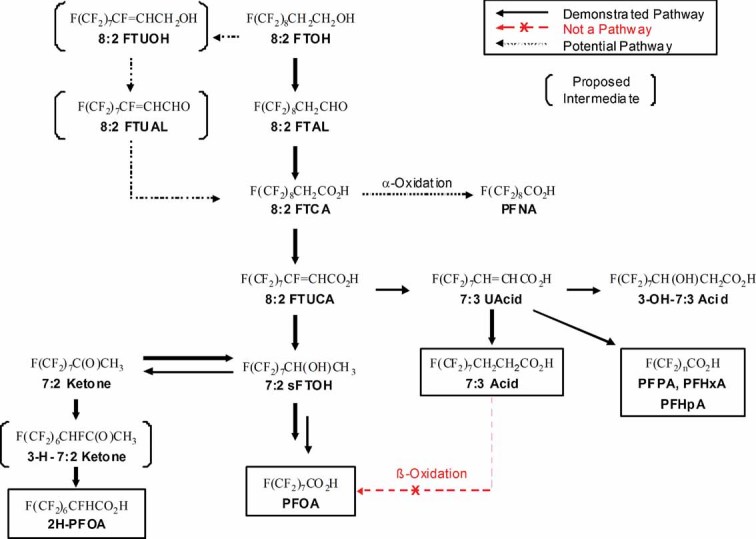
Aerobic biotransformation pathways for 8:2 fluorotelomer alcohol (8:2 FTOH). Adapted from Wang et al. (2009).

The aerobic biodegradation and metabolic degradation pathways for fluorotelomer alcohols have been well studied (Frömel and Knepper 2010). A general overview of the 8:2 FTOH aerobic biodegradation pathways is presented in Figure 5. The pathways and yields of transformation products depend on the matrix in which the environmental microbial degradation (e.g., sludge, soil) or metabolism (rat, mouse, in vivo, in vitro) takes place and the length of the perfluoroalkyl chain in the fluorotelomer alcohol (Hagen et al. 1981; Dinglasan et al. 2004; Martin et al. 2005; Wang et al. 2009; Butt et al. 2010a; Liu et al. 2010; Brandsma et al. 2011). In general, the first step in biodegradation is aerobic oxidation of the starting n:2 fluorotelomer alcohol to form the corresponding n:2 fluorotelomer aldehyde, C_n_F_2n+1_CH_2_CHO (n:2 FTAL; Table 3), a short-lived, highly reactive species. The aldehyde is rapidly oxidized to form the corresponding n:2 fluorotelomer carboxylic acid, C_n_F_2n+1_CH_2_COOH (n:2 FTCA; Table 3). Next, dehydrohalogenation of the acid occurs to form the corresponding n:2 unsaturated carboxylic acid, C_n−1_F_2n−1_CF=CHCOOH (n:2 FTUCA; Table 3). The dehydrohalogenation of the starting n:2 fluorotelomer alcohol to form the n:2 unsaturated fluorotelomer alcohol, C_n−1_F_2n−1_CF=CHCH_2_OH (Table 3), and oxidation to yield the n:2 unsaturated fluorotelomer aldehyde, C_n−1_F_2n−1_CF=CHCHO (n:2 FTUAL; Table 3), have also been observed. Thereafter, a host of transient and stable transformation products, including PFCAs, have been identified. A unique transformation product identified is a polyfluorinated carboxylic acid with the same number of total C atoms as the parent n:2 FTOH where the 2 F atoms of the -CF_2_- group directly adjacent to the -CH_2_CH_2_- moiety have been replaced with H atoms, C_n−1_F_2n−1_CH_2_CH_2_COOH, and a corresponding unsaturated acid, C_n−1_F_2n−1_CH=CHCOOH (Table 3) (Martin et al. 2005; Wang et al. 2005; Fasano et al. 2006; Wang et al. 2009; Butt et al. 2010a). For these substances, we suggest for simplicity that either the formal name of the acid be used or the simple acronyms *x*:3 Acid and *x*:3 UAcid, where the *x* (= n − 1) designates the number of perfluorinated carbons and “3” the number of nonfluorinated C atoms. For the remaining transformation products, we suggest adopting the naming given to these substances by the authors (e.g., Martin et al. 2005; Wang et al. 2009; Butt et al. 2010a; Liu et al. 2010). In a sediment–water microcosm, the degradation products observed from n:2 FTCA substrates were the corresponding PFCAs, whereas n:2 FTUCAs also led to (n − 1):3 Acids (Myers and Mabury 2010).

In mammals, the metabolic pathways for 8:2 and 6:2 FTOHs have been well studied in vivo in rats and mice and in vitro in rats, mice, and human hepatocytes. In general, the majority of administered FTOH test substance was eliminated rapidly in urine as conjugates. Absorption, distribution, metabolism, and elimination (ADME) studies using [^14^C]-radiolabeled FTOHs have been conducted. The characteristic degradation products observed in microbial studies, including PFCAs, as well as some of their conjugates, have been reported in urine and at trace levels in organs and tissues (Fasano et al. 2006; Nabb et al. 2007; Fasano et al. 2009). The reader is referred to the articles for greater detail on these studies.

**Figure 6:**
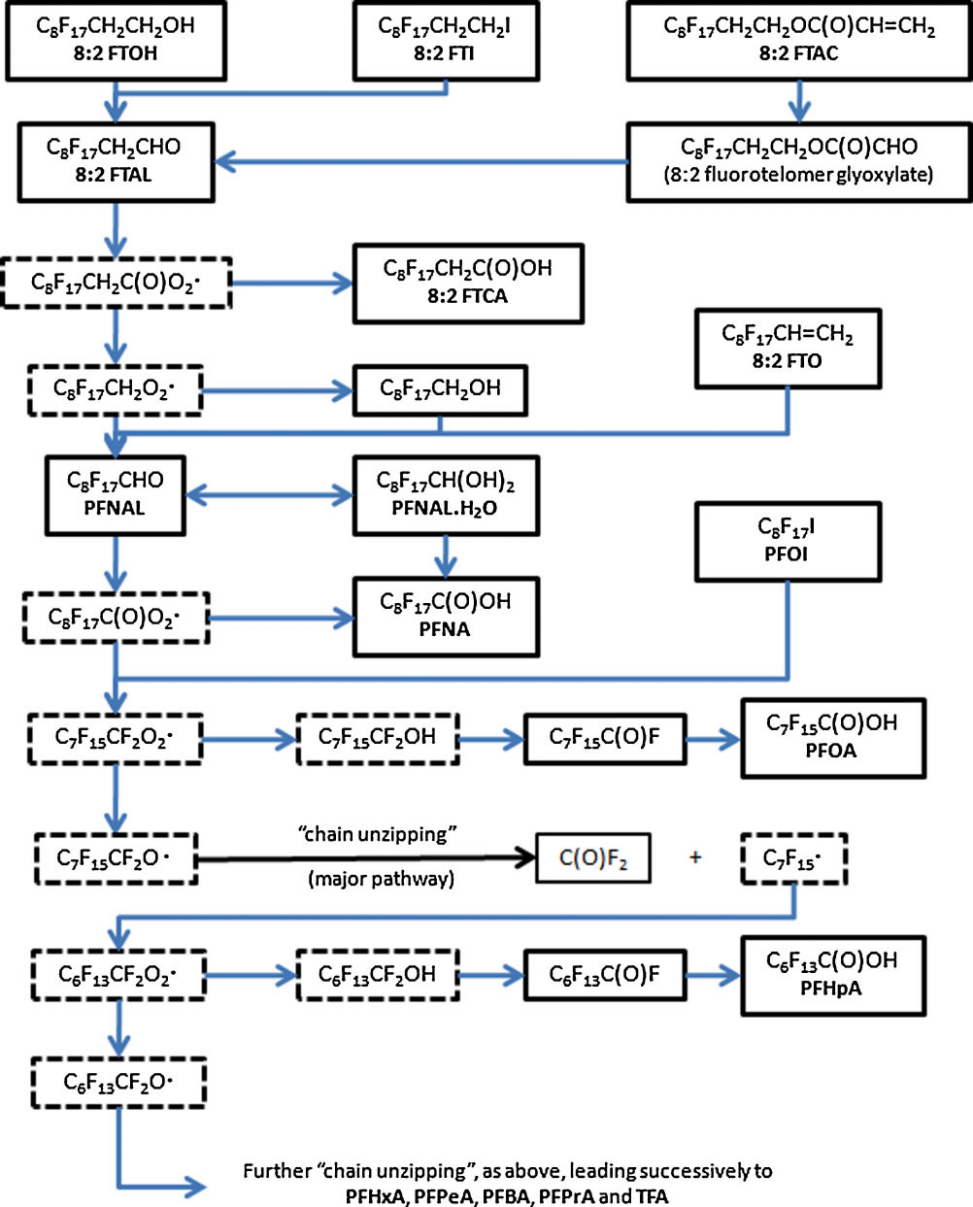
Simplified atmospheric degradation scheme for 8:2 fluorotelomer derivatives. Free-radical and transient molecular intermediates are shown in boxes with a dashed outline, while the starting compounds, the more stable molecular intermediates, and the final products are shown in boxes with a solid outline, their acronyms being indicated in bold type. An arrow on the chart often implies several elementary steps: i.e., certain intermediates are omitted.

In atmospheric degradation studies, reviewed by Young and Mabury (2010), it has been shown that oxidation of n:2 FTOHs also leads to the formation of n:2 FTALs, n:2 FTCAs, and perfluoroalkyl aldehydes, C_n_F_2n+1_CHO (PFALs; Table 2). Low yields (typically 1%–5%) of PFCAs having the same number of perfluorinated C atoms as the parent FTOH, or fewer, down to CF_3_COOH, may be expected in low-NO_x_ atmospheres. The PFCAs with n − 2 or fewer perfluorinated C atoms result from “unzipping” of the perfluoroalkyl chain, by splitting off of C(O)F_2_ molecules from the intermediate perfluoroalkoxy radicals (Ellis et al. 2004). Nevertheless, complete mineralization to C(O)F_2_ is the major atmospheric outcome, and the yields of PFCAs decline as atmospheric NO_x_ levels increase (Ellis et al. 2004; Wallington et al. 2006; Young and Mabury 2010). A simplified scheme, given in Figure 6, shows the key intermediates in the atmospheric degradation of n:2 FTOHs to the products mentioned above, illustrated for *n* = 8. This scheme also includes the atmospheric breakdown pathways for FTIs and FTOs, discussed above, as well as for FTACs (Butt et al. 2009), because all these fluorotelomer derivatives have part of their degradation mechanism in common. This is also likely to be the case for PFAIs (Figure 6), assuming they photolyze easily to perfluoroalkyl radicals (which add O_2_ to give perfluoroalkylperoxy radicals) in the lower atmosphere, as has been demonstrated for CF_3_I (Solomon et al. 1994).

It is worth noting here that the PFALs will probably exist in cloud and surface waters largely as their *gem*-diol hydrates, C_n_F_2n+1_CH(OH)_2_ (PFAL·H_2_Os; Table 2), unlike the FTALs for which the hydration equilibrium is much less favorable (Rayne and Forest 2010b). With estimated p*K*_a_ values of 9 or higher, the PFAL·H_2_Os will not be ionized to any great extent under environmental conditions, whereas the corresponding hydrates formed from FTALs are even weaker acids (p*K*_a_ > 12) (Rayne and Forest 2010b).

The esters of FTOHs may hydrolyze abiotically or biotically to FTOHs and, hence, ultimately lead to the same range of fluorinated transformation products described above. Hydrolysis studies of mono- and polyesters and monourethanes containing a fluorotelomer moiety have recently been reported (Dasu et al. 2010). Moreover, as expected, characteristic FTOH degradation products were detected when rainbow trout were exposed to 8:2 FTAC through their diet (Butt et al. 2010b), and when rats were dosed with monoPAPs or diPAPs (D'eon and Mabury 2007, 2011). Both FTOHs and their transformation products were observed in experiments intended to simulate aerobic biodegradation of monoPAPs and diPAPs in WWTPs (Lee et al. 2010). The abiotic hydrolysis of FTACs has been predicted to have half-lives of years in marine systems but possibly only days in landfills (Rayne and Forest 2010c). Hydrolytic stability studies, conducted under OECD 111 Guidelines, on a commercial fluorotelomer-based acrylate polymer (Russell et al. 2008) and a urethane polymer (Russell et al. 2010) showed no discernible hydrolysis. Nevertheless, there is much debate regarding the hydrolysis and biodegradation of commercial fluorotelomer-based polymers (Russell et al. 2008; Koch et al. 2009; Russell et al. 2009; Washington et al. 2009a; Washington et al. 2009b) that future research will illuminate.

A number of reported observations of n:2 FTCAs and/or n:2 FTUCAs have occurred in environmental media and biota such as atmospheric particles (Stock et al. 2007), indoor dust (Barber et al. 2007), precipitation (Loewen et al. 2005; Scott et al. 2006; Taniyasu et al. 2008; Kwok et al. 2010; Scott et al. 2010), surface waters (Stock et al. 2007; Ahrens et al., 2009a; Scott et al. 2010; Zushi et al. 2011), sediments (Stock et al. 2007), WWTP effluent (Sinclair and Kannan 2006; Zushi et al. 2011), sewage sludge (Zhang et al. 2010), landfill leachate (Huset et al. 2011), animal biota (Houde et al. 2005; Taniyasu et al. 2005; Butt, Mabury, et al. 2007; Butt, Muir, et al. 2007; Furdui et al. 2007; Gebbink et al. 2009), human breast milk (So et al. 2006), and foodstuffs (Ostertag et al. 2009). The 7:3 Acid has also been detected in biota (Powley et al. 2008; Peng et al. 2010; Guruge et al. 2011). The various perfluorinated and polyfluorinated aldehydes discussed above have apparently not yet been found in environmental samples. This is most likely due to their highly reactive nature, because only trapping experiments have qualified their presence thus far in laboratory studies.

##### Fluorotelomer sulfonic acids

The n:2 fluorotelomer sulfonic acids, C_n_F_2n+1_CH_2_CH_2_SO_3_H (FTSAs; Table 3) have been found in groundwater, soil, and biota, especially at military bases, firefighting training sites, and locations where major fires have been extinguished through use of AFFFs (Schultz et al. 2004; Norwegian Pollution Control Authority 2008; Oakes et al. 2010). They have also been detected in WWTP effluents (Huset et al. 2008; Ahrens et al., 2009b), landfill leachate (Eggen et al. 2010; Huset et al. 2011), precipitation and fresh surface waters (Kim and Kannan 2007; Scott et al. 2010; Nguyen et al. 2011), seawater contaminated by AFFFs (Taniyasu et al. 2005), sediments (Zushi et al. 2010), Arctic biota (Miljeteig et al. 2009), and human serum (Lee and Mabury 2011). These FTSAs arise from the degradation of more complex fluorotelomer-based substances containing the C_n_F_2n+1_CH_2_CH_2_S–R or C_n_F_2n+1_CH_2_CH_2_SO_2_–R moiety (where R is a hydrophilic functional group that provides surfactant properties). These precursor compounds may be used as components of firefighting foams (Bertocchio and Foulletier 1970; Falk 1982; Schultz et al. 2004), e.g., the betaine 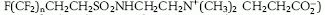, or in food packaging applications, e.g., the fluororotelomer mercaptoalkyl phosphate esters (Lee and Mabury 2011; Trier, Granby, et al. 2011; Trier, Nielsen, et al. 2011). FTSAs have been shown to undergo slow aerobic biotransformation to form trace levels of PFCAs (Wang et al. 2011). It should be noted that 6:2 FTSA has been referred to in some literature as “tetrahydro PFOS.” Because 6:2 FTSA is both chemically and biologically very different from PFOS (Wang et al. 2011), we strongly discourage this usage and recommend 6:2 FTSA be used in naming this substance.

#### Perfluoroalkane sulfonamido derivatives: Perfluoroalkane sulfonamides, sulfonamidoethanols, sulfonamidoethyl acrylates, and sulfonamidoethyl methacrylates

**Figure 7:**
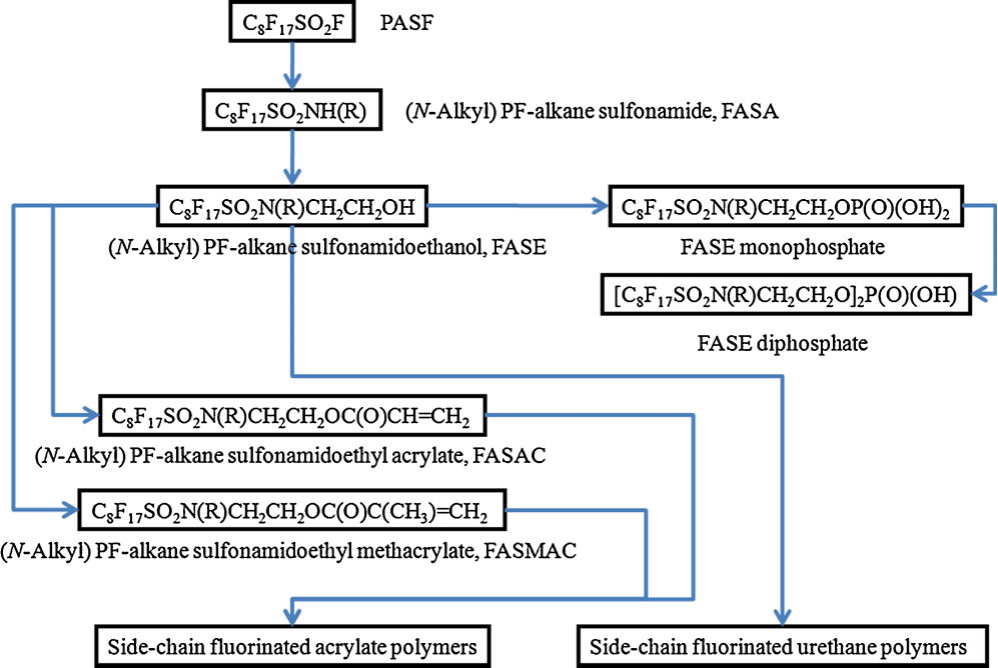
Perfluoroalkane sulfonamido derivatives synthesized from perfluoroalkane sulfonyl fluorides (PASFs), exemplified for a starting PASF with 8 C atoms. N.B. Names and acronyms for substance families are indicated. Those for the specific compounds shown can be found in the Supplemental Data.

In the same way as the perfluoroalkyl iodides and fluorotelomer iodides are important building blocks for a broad range of fluorotelomer derivatives, the perfluoroalkane sulfonyl fluorides, C_n_F_2n+1_SO_2_F (PASFs; Table 2) play an analogous role as precursors in the manufacture not only of the PFSAs already discussed, but also of a variety of compounds containing the perfluoroalkane sulfonamido group, C_n_F_2n+1_SO_2_N< (Tables 2 and 3). This is illustrated in Figure 7 for the synthesis of several families of perfluoroalkane sulfonamido derivatives, exemplified for a starting PASF with 8 C atoms. PFSAs were directly manufactured by hydrolysis of PASFs and the various salt forms (ammonium, diethanolamine, and K and Li salts) were manufactured by neutralization of the acids. The greater part of the production of PASFs (notably POSF), however, was used to produce fluorinated surfactants and high-molecular-weight fluorinated polymeric products (3M Company 1999). The major pathway for conversion of PASFs into commercial derivatives involves reacting them in a first step with a primary amine, generally methylamine or ethylamine, to give *N*-methyl or *N*-ethyl perfluoroalkane sulfonamides, C_n_F_2n+1_SO_2_NH(C_m_H_2m+1_), where *m* = 1 or 2 (MeFASAs and EtFASAs; Table 3) (3M Company 1999; Lehmler 2005). These *N*-alkyl FASAs are, in some cases, commercial products in their own right, as well as building blocks for further synthesis. For instance, *N*-ethyl perfluorooctane sulfonamide, C_8_F_17_SO_2_NH(C_2_H_5_), or EtFOSA, is the pesticide sulfluramid. In a 2nd major industrial reaction step, *N*-alkyl FASAs are reacted with ethylene carbonate to give another series of building blocks, the *N*-methyl or *N*-ethyl perfluoroalkane sulfonamido ethanols, C_n_F_2n+1_SO_2_N(C_m_H_2m+1_)CH_2_CH_2_OH, where *m* = 1 or 2 (MeFASEs and EtFASEs; Table 3) (3M Company 1999; Lehmler 2005). These *N*-alkyl FASEs are analogous to FTOHs. Because they are alcohols, they can be converted into acrylates and methacrylates, as well as into phosphates and other derivatives (3M Company 1999) that will not be discussed further here. The *N*-alkyl perfluoroalkane sulfonamidoethyl acrylates, C_n_F_2n+1_SO_2_N(C_m_H_2m+1_)CH_2_CH_2_OC(O)CH=CH_2_, where *m* = 1 or 2 (MeFASACs and EtFASACS; Table 3) and the corresponding *N*-alkyl perfluoroalkane sulfonamidoethyl methacrylates, C_n_F_2n+1_SO_2_N(C_m_H_2m+1_)CH_2_CH_2_OC(O)C(CH_3_)=CH_2_ (MeFASMACs and EtFASMACs; Table 3) are used in a similar manner to the fluorotelomer acrylates and methacrylates, as comonomers for synthesizing acrylic polymers used in surface protection applications (3M Company 1999).

The (alkyl-)FASA, FASE, FASAC, and FASMAC intermediates were the principal building blocks of many fluorochemical products used in surface treatments, paper packaging protectors, and other specialist applications. A more extensive range of commercial compounds has, however, been produced, as described in industry reports (3M Company 1999). In 2002, the largest historic manufacturer of perfluorooctane sulfonyl derivatives (*n* ≥ 6) ceased manufacture (3M Company 2000a; USEPA 2000) and has since introduced products based on perfluorobutane sulfonyl chemistry (Renner 2006; Ritter 2010). Meanwhile, existing and new manufacturers continue to make PFOS and other long-chain perfluoroalkane sulfonates and products derived from them.

##### Degradation products of perfluoroalkane sulfonamido derivatives

**Figure 8:**
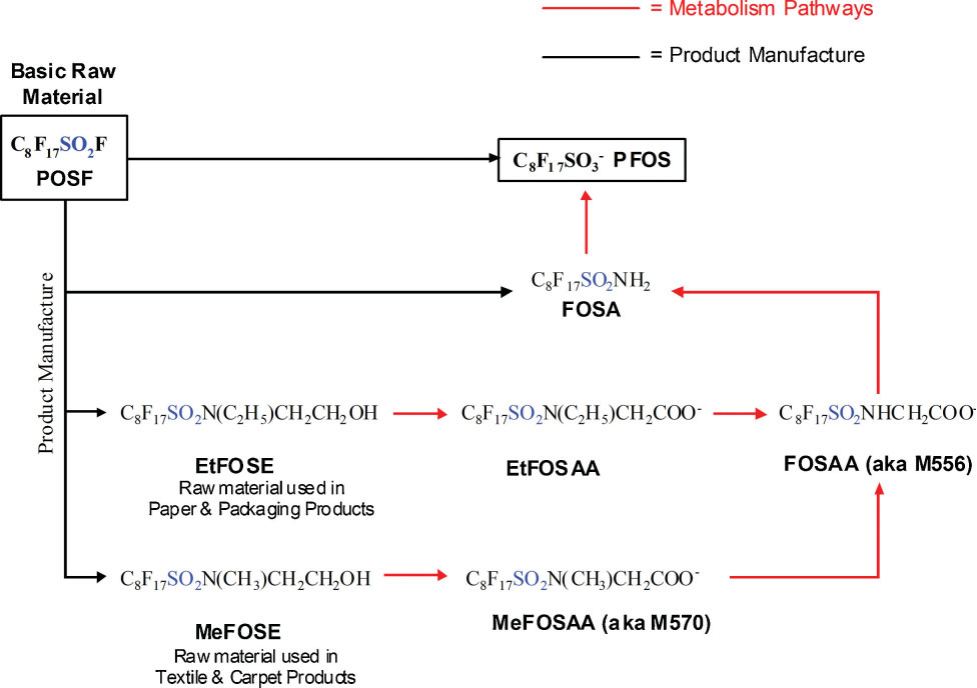
Transformation pathways for perfluoroalkane sulfonamido derivatives. Adapted from Olsen et al. 2002 and Olsen et al. 2005.

Published studies on the aerobic biotransformation of the perfluoroalkane sulfonamido derivatives focus on those compounds having 8 perfluorinated C atoms, in particular *N*-ethyl perfluorooctane sulfonamidoethanol (EtFOSE), which is ultimately degraded to PFOS. Various intermediates leading to this perfluoroalkane sulfonic acid have been reported, including the members of the following families (Tables 2 and 3) with *n* = 8: *N*-ethyl perfluoroalkane sulfonamidoacetic acids (EtFASAAs), C_n_F_2n+1_SO_2_N(C_2_H_5_)CH_2_COOH; *N*-ethyl perfluoroalkane sulfonamides (EtFASAs), C_n_F_2n+1_SO_2_NH(C_2_H_5_); perfluoroalkane sulfonamidoacetic acids (FASAAs), C_n_F_2n+1_SO_2_NHCH_2_COOH; perfluoroalkane sulfonamides (FASAs), C_n_F_2n+1_SO_2_NH_2_; FASA *N*-glucuronides, and perfluoroalkane sulfinic acids (PFSIAs), C_n_F_2n+1_SO_2_H (Lange 2000, 2001; Tomy, Tittlemier, et al. 2004; Xu et al. 2004; Boulanger et al. 2005; Xu et al. 2006; Rhoads et al. 2008; Xie et al. 2009) (Figure 8). There appears to be conflicting evidence as to whether PFOA can be formed in the environment from EtFOSE as a minor end product (Lange 2001; Tomy, Tittlemier, et al. 2004; Boulanger et al. 2005; Rhoads et al. 2008).

The *N*-alkyl perfluoroalkane sulfonamidoethyl acrylates and methacrylates, and polymers based on them, may undergo hydrolysis of the ester linkage in the environment to give *N*-alkyl FASEs (Martin et al. 2010) and, hence, lead to the same perfluoroalkyl biotransformation products. However, there do not appear to have been any published experimental studies that explicitly demonstrate this to be the case.

Studies on the hydroxyl-radical–initiated degradation of EtFOSE in the aqueous phase show that some of the intermediates and products observed, including EtFOSAA, EtFOSA, FOSAA, FOSA, and PFOA, are the same as those reported for biodegradation. On the other hand, PFOS and PFOSI were not observed or were present at only trace levels in these abiotic studies (Hatfield 2001; Plumlee et al. 2009) and FOSA was considered to be a stable end product (Plumlee et al. 2009).

Atmospheric degradation pathways have been studied for 2 perfluoroalkane sulfonamido derivatives having 4 perfluorinated C atoms. The breakdown of EtFBSA, C_4_F_9_SO_2_NH(C_2_H_5_), has been shown to proceed through ketone and aldehyde intermediates to give PFCAs, i.e., PFBA, PFPrA and TFA, as well as COF_2_ (Martin et al. 2006). The PFPrA and TFA are formed via chain unzipping of the perfluoroalkoxy radical, as already mentioned above for FTOHs and depicted schematically on Figure 6, so that alkyl-FASAs share part of their degradation scheme with FTOHs. PFBS was not observed to be formed from EtFBSA (Martin et al. 2006). MeFBSE, C_4_F_9_SO_2_N(CH_3_)CH_2_CH_2_OH, was observed to degrade to the same PFCAs as EtFBSA, together with PFBS, MeFBSA, and other products (D'eon et al. 2006).

##### Environmental occurrence of perfluoroalkyl sulfonamido derivatives

Various perfluoroalkyl sulfonamido derivatives have been found in the environment and human samples, whether this is due to industrial or consumer use of these compounds as such, losses during manufacturing operations, presence as “residuals” in other commercial products, or formation as environmental degradation products or metabolites of precursors.

It should be noted that perfluoroalkane sulfonamido derivatives bearing a H on the N atom are acidic in nature and can dissociate to an amide anion, to a greater or lesser extent depending on the ambient environmental or physiological conditions, with the degree of branching of the perfluoroalkyl chain having a significant influence on the p*K*_a_ for a given family of compounds (Rayne and Forest 2009a). For FASAAs, there is the additional possibility of dissociation of the carboxylic H (more acidic than the amide H), whereas for the *N*-alkyl FASAAs, this is the only possible ionization (Rayne and Forest 2009a). The dissociated species are not depicted in the list of compounds provided in the Supplemental Data.

All the families of perfluoroalkane sulfonamido derivatives discussed above and depicted in Tables 2 and 3 have been found in the environment or in human biota. Those with 8 perfluorinated C atoms are, in general, much more abundant than those with other chain lengths. However, more recently, compounds with 4 such C atoms have also been reported. The medium in which they are detected depends on their physical properties and on their likelihood of being formed there from precursors. In atmospheric air and its associated particulate matter, commonly detected compounds are the relatively volatile FOSA, MeFBSA, MeFOSA, Me_2_FOSA, EtFOSA, MeFBSE, EtFBSE, MeFOSE, and EtFOSE (Martin et al. 2002; Barber et al. 2007; Piekarz et al. 2007; Stock et al. 2007; Dreyer, Matthias, et al. 2009; Dreyer, Weinberg, et al. 2009; Dreyer et al. 2010; Langer et al. 2010; Shoeib et al. 2010; Haug et al. 2011; Weinberg et al. 2011a, 2011b), whereas house dust has been found to contain FOSA, MeFOSA, EtFOSA, MeFOSE, EtFOSE (Shoeib et al. 2005; Kato et al. 2009; Goosey and Harrad 2011), the acrylate MeFOSAC (Shoeib et al. 2005), and the oxidation products MeFOSAA and EtFOSAA (Kato et al. 2009). FOSA has also been detected in open ocean water, sometimes at levels comparable to those of PFOA (Ahrens, Gerwinski, et al. 2010; Ahrens, Xie, et al. 2010; Busch et al. 2010b; Kirchgeorg et al. 2010), as well as in precipitation (Kim and Kannan 2007; Taniyasu et al. 2008; Kwok et al. 2010), river and lake water (Kim and Kannan 2007; So et al. 2007; Ahrens et al. 2009b; Ahrens, Gerwinski, et al. 2010; Scott et al. 2010; Zushi et al. 2011), groundwater (Murakami, Kuroda, et al. 2009), surface runoff water (Kim and Kannan 2007; Murakami, Shinohara, et al. 2009), landfill leachate (Kallenborn et al. 2004; Busch et al. 2010a; Huset et al. 2011), sewage sludge (Llorca et al. 2011), and drinking water (Ericson et al. 2009). In wildlife, FOSA is often the predominant sulfonamido species, although it is generally present at lower levels than PFOS (Sturm and Ahrens 2010 and references therein), whereas EtFOSA and/or Et_2_FOSA (Tomy, Budakowski, et al. 2004; Tittlemier et al. 2005; Tittlemier et al. 2006; Löfstrand et al. 2008; Ahrens, Siebert, et al. 2009; Yeung et al. 2009), MeFOSE (Ahrens and Ebinghaus 2010), FOSAA (Peng et al. 2010), and EtFOSAA (Yoo et al. 2009) have also been reported. FOSA and various *N*-alkyl-FOSAs (Me-, Et-, Me_2_-, and Et_2_-FOSAs) were detected in foodstuffs (Tittlemier et al. 2005; Tittlemier et al. 2006). WWTP effluents and river, coastal, and ocean waters were found to contain some *N*-alkyl sulfonamido derivatives (MeFBSA, MeFBSE, MeFOSE, EtFOSE, MeFBSAA, MeFOSAA, and EtFOSAA) as well as FOSA and FOSAA (Ahrens et al. 2009a; Ahrens et al. 2009b; Ahrens, Gerwinski, et al. 2010; Huset et al. 2011; Nguyen et al. 2011; Zushi et al. 2011). In human blood, the sulfonamido derivatives FOSA, FOSAA, MeFOSAA, and EtFOSAA have been quantified (Kannan et al. 2004; Calafat et al. 2007; Olsen et al. 2008; Weihe et al. 2008; Toms et al. 2009; Lee and Mabury 2011). MeFOSAA and/or EtFOSAA have also been detected in precipitation (Taniyasu et al. 2008; Kwok et al. 2010), wildlife (Yoo et al. 2009), sediments (Higgins et al. 2005; Ahrens, Taniyasu, et al. 2010; Zushi et al. 2010) and WWTP influent and effluent (Boulanger et al. 2005). These 2 compounds have also been shown to be among the most abundant PFAS components of municipal WWTP sludge (Higgins et al. 2005; Sepulvado et al. 2011), in which FOSAA has also been detected (Higgins et al. 2005).

#### Perfluoroalkyl and polyfluoroalkyl ether carboxylic acids

Salts of perfluoroalkyl ether carboxylic acids (not depicted in the tables) and polyfluoroalkyl ether carboxylic acids (Table 3) are widely cited in patents as alternative fluoropolymer processing aids, that are more environmentally and/or toxicologically acceptable alternatives to APFO and APFN. A common feature is that a terminal –COO^−^ group, attached to one or both ends of the fluorinated ether chain, is the common hydrophile, generally with an 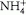 counter-ion (Tsuda et al. 2003; Visca et al. 2003; Higuchi et al. 2005; Hintzer et al. 2005; Brothers et al. 2008; Ishikawa et al. 2008; Gordon 2011). These and/or other alternative surfactants are expected to enable manufacturers to meet the USEPA 2010/15 Stewardship Program goal to eliminate the use of PFOA and higher homologues. Most recently, a toxicological evaluation for one of these substances (ammonium 4,8-dioxa-3*H*-perfluorononanoate; Table 3) has been published (Gordon 2011). Substances based on certain members of this family of compounds have a sufficient number of repeating units (together with other characteristics) to enable them to be considered to be polymers under the European Union REACH legislation (ECHA 2008).

### Fluorinated polymers

The polymers discussed in this section are those: 1) whose synthesis involves the incorporation of one or more PFASs as monomers. In this case, there is some potential (theoretical or demonstrated) for the degradation of the polymer, during or after its useful lifetime, to lead to release of PFASs to the environment; or 2) whose manufacture requires the use of a PFAS as a processing aid.

#### Fluoropolymers

Fluoropolymers contain F bound to one or both of the olefinic C atoms, to form a perfluorinated C-only polymer backbone with F atoms directly attached to it (Table 4). Examples of fluoropolymers are polytetrafluoroethylene (PTFE); polyvinylidene fluoride (PVDF); polyvinyl fluoride (PVF); copolymers of tetrafluoroethylene (TFE) and hexafluoropropylene (HFP); terpolymers of TFE, vinylidene fluoride, and HFP; and copolymers of TFE and ethylene. Certain grades of fluoropolymers, manufactured by emulsion (or dispersion) polymerization, in order to obtain a fine particle size distribution, require the use of a fluorosurfactant “processing aid.” This additive, used at a level of a few tenths of a percent relative to the amount of polymer produced (Prevedouros et al. 2006), was often traditionally the ammonium salt of PFOA or PFNA. The fluorosurfactant is removed when the fluoropolymer aqueous emulsion is dried for sale as a solid. Similarly, when an aqueous fluoropolymer emulsion is used, the polymer is heated to cure it. High cure temperatures thermally destroy the fluorosurfactant. At low cure temperatures, residual surfactant may remain (Guo et al. 2009). Most producers have discontinued the use of PFOA and PFNA salts as processing aids and have developed and implemented more environmentally acceptable alternatives, as discussed above in the *Perfluoroalkyl and Polyfluoroalkyl Ether Carboxylic Acids* section. It should be emphasized that those grades of fluoropolymers (e.g., PTFE, PVDF) that are made by suspension (rather than emulsion) polymerization do not require a fluorosurfactant to be used as a “processing aid.”

#### Perfluoropolyethers

Perfluoropolyethers (PFPEs; Table 4) are polymers in whose backbone -CF_2_-, -CF_2_CF_2_-, and possibly -CF(CF_3_)CF_2_- units are separated by O atoms. For example, the ultraviolet-initiated copolymerization of TFE with O_2_ leads to PFPEs with a structure that may be represented symbolically by CF_3_O(CF_2_CF_2_O)_m_(CF_2_O)_n_CF_3_, although this overall formula does not show that the -CF_2_O- and -CF_2_CF_2_O- units are generally distributed randomly rather than in blocks (Sianesi et al. 1994). If the photopolymerization is conducted using hexafluoropropylene (HFP) instead of (or together with) TFE, then PFPEs with the overall formula CF_3_O(CF_2_CF_2_O)_m_(CF_2_O)_n_[CF(CF_3_)CF_2_O]_p_CF_3_ are obtained. Furthermore, the PFPE -[CF(CF_3_)CF_2_O]_n_- can be synthesized by homopolymerization of HFP (ep)oxide.

Because the repeating units of these PFPEs contain only 2 or 3 perfluorinated C atoms per O atom, their degradation cannot lead to the formation of long-chain PFCAs. The reason for mentioning them in this review is that certain difunctional polymeric perfluoro-polyether products, corresponding to the overall formula X-CF_2_O(CF_2_CF_2_O)_m_(CF_2_O)_n_CF_2_–X, where X is a hydrophilic group, are marketed as surface treatments for natural stone, metal, glass, plastic, textiles, leather, and paper and paperboard treatment for food-contact applications. These functionalized PFPEs bring properties such as a low surface energy, high contact angle, reduced coefficient of friction, and high oleo-hydrophobicity (Solvay Solexis 2011), so that they are potential alternatives to the ECF-based polymers, fluorotelomer-based polymers, and fluorinated oxetane polymers described in this review.

#### Side-chain–fluorinated polymers

In contrast to the polymers described previously, side-chain–fluorinated polymers do not have perfluorinated or polyfluorinated polymer backbones, but are composed of variable composition backbones with polyfluoroalkyl (and possibly perfluoroalkyl) side chains (Table 4). With regard to the sources of long-chain PFAAs, we review 3 groups of side-chain–fluorinated polymers distinguished from one another by the linkage (acrylate and/or methacrylate, urethane, and oxetane) between the polymer backbone and the polyfluoroalkyl (and possibly perfluoroalkyl) side chains. Side chains of each of these polymer types may possess the ability to sever from the polymer chain to become PFASs shown in Tables 2 and 3. It should be noted, however, that this transformation process can occur over long time periods (e.g., >1000 y) and may exhibit low yields of PFASs such that their contribution to the environmental inventory of long-chain PFAAs may be insignificant relative to other historical and current sources. Further research is required to clarify this question.

##### Fluorinated acrylate polymers

Fluorinated acrylate polymers are made by polymerizing a fluorinated acrylate (or methacrylate) monomer, in which the alcohol moiety is n:2 FTOH, C_n_F_2n+1_CH_2_CH_2_OH, or an alkyl-FASE, C_n_F_2n+1_SO_2_N(R)CH_2_CH_2_OH, where R = CH_3_, C_2_H_5_, or another alkyl group (Table 4). Some possible structures for the fluorinated acrylate monomers are therefore:


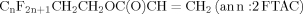



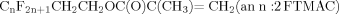



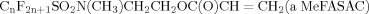



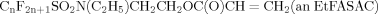



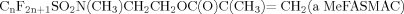



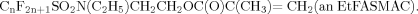


These fluorinated acrylate monomers are copolymerized with one or more nonfluorinated acrylate monomers, and possibly other monomers, to give the final side-chain fluorinated acrylate polymers. These types of polymers are useful as water-, stain- and grease-proofing finishes for textile, leather, and paper surfaces. As stated above, it is not yet clear to what extent such polymers may break down in the environment to give PFAAs, such as PFOA, PFOS, PFBA, and PFBS. Moreover, although we have shown only fluorotelomer and perfluoroalkane sulfonamido (meth)acrylates, the term “side-chain–fluorinated polymer” would encompass many other potential structures and products therefrom that conform to the definition provided.

##### Fluorinated urethane polymers

Polymeric materials for repelling water and stains may also be based on urethane polymers formed by reacting fluorotelomer alcohols (FTOHs), or perfluoroalkane sulfonamidoethanols (alkyl-FASEs), with polyisocyanato homopolymers, followed by a cross-linking step (Kirchner 1989). The products are polyfluorinated in their side chains (Table 4). They are used mainly in textile applications. In the case of an (8:2) FTOH-based urethane polymer, a recent study has shown that the half-life with respect to biodegradation to PFOA in aerobic soils is on the order of a century (Russell et al. 2010).

##### Fluorinated oxetane polymers

**Figure 9:**
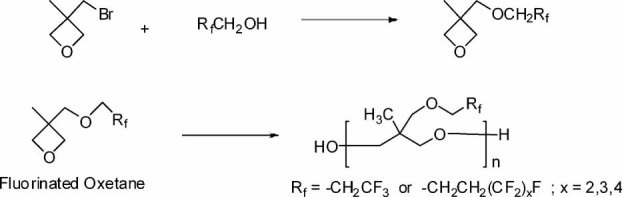
Oxetane-based fluorinated polymers.

An alternative fluorinated polymer technology to those described thus far originates from the reaction of polyfluorinated alcohols with oxetanes bearing a -CH_2_Br group in their side chains, to create oxetane monomers that can undergo ring-opening polymerization to give side-chain–polyfluorinated polyethers (Figure 9). These fluorinated oxetane polymers (Table 4) are offered in many forms and functionalities primarily as fluorosurfactants and coatings additives (Kausch et al. 2002; Kausch et al. 2003a, 2003b; Thomas 2006; Omnova Solutions 2011).

#### Commercial articles containing multiple types of fluorinated polymers

It should be noted that there are commercial products that contain both fluoropolymers and side-chain–fluorinated polymers, which can cause confusion about the origin of individual PFASs. In all-weather clothing products, for example, multiple layered materials containing different types of polymers are common. A porous PTFE membrane layer is often used in garments to make the fabric “breathable.” The outer fabric layer may be nylon or polyester treated with a side-chain–fluorinated polymer water repellent. Analyses of all-weather clothing revealed the presence of FTOHs in the outer layer of some all-weather clothing products (Berger and Herzke 2006; Schulze and Norin 2006). The origin of the FTOHs is not the PTFE breathable membrane.

## SUMMARY AND FUTURE PROSPECTS

We have provided an overview of PFASs detected in the environment, wildlife, and humans and recommended clear, specific, and descriptive terminology, names, and acronyms for PFASs. We hope the terminology will be widely adopted and used. Future interest in fluorinated substances by the global scientific community is expected to remain high, and continued publications should be numerous. The consistent use of the terminology described here by this community will facilitate clear and coherent communication, understanding, interpretation, and comparison of published studies as well as serve to highlight similarities and acknowledge key differences between PFASs. We strongly discourage the use of broad, poorly defined terms and acronyms in favor of the clear, specific, and descriptive terminology provided here.

## SUPPLEMENTAL DATA

**Supplemental Data.** Names, formulas, acronyms, and CAS numbers for selected perfluoroalkyl and polyfluoroalkyl substances. Terminology decision flow charts.
