# Supplementary material for: Perfluoroalkyl and Polyfluoroalkyl Substances in the Environment: Terminology, Classification, and Origins
Source: Integr Environ Assess Manag. 2011 Jul 25;7(4):513–41. doi: 10.1002/ieam.258 (PMC3214619; doi:10.1002/ieam.258)
Supplement: Supplementary file 1 [file ieam0007-0513-SD1.doc]

**SUPPLEMENTAL DATA**

**Perfluoroalkyl and PolyfluorOalkyl Substances in the Environment: Terminology, Classification, and Origins**

Robert C. Buck†, James Franklin*‡, Urs Berger§, Jason M. Conder||, Ian T. Cousins§, Pim de Voogt#, Allan Astrup Jensen††, Kurunthachalam Kannan‡‡, Scott A. Mabury§§, and Stefan P. J. van Leeuwen||||

†E.I. du Pont de Nemours & Co. Inc., DuPont Chemicals and Fluoroproducts, 4417 Lancaster Pike, CRP 702-2211B, Wilmington, DE 19880-0702 USA ([Robert.C.Buck@USA.dupont.com](mailto:Robert.C.Buck@USA.dupont.com))

‡CLF-Chem Consulting SPRL, 3 Clos du Châtaignier, BE-1390 Grez-Doiceau, Belgium ([james.franklin@skynet.be](mailto:james.franklin@skynet.be))

§Department of Applied Environmental Science (ITM), Stockholm University, Svante Arrhenius väg 8, SE-10691 Stockholm, Sweden ([urs.berger@itm.su.se](mailto:urs.berger@itm.su.se); [ian.cousins@itm.su.se](mailto:ian.cousins@itm.su.se))

||ENVIRON International Corporation, 18100 Von Karman Avenue, Suite 600, Irvine, CA 92612, USA ([jconder@environcorp.com](../../../../C:%5CUsers%5CCLF-Chem%20Consulting%5CAppData%5CLocal%5CMicrosoft%5CWindows%5CTemporary%20Internet%20Files%5CContent.Outlook%5CXO8LXUG3%5Cjconder@environcorp.com))

#Institute for Biodiversity and Ecosystem Dynamics, University of Amsterdam, PO Box 94248, NL-1090 GE Amsterdam, The Netherlands ([w.p.devoogt@uva.nl](mailto:w.p.devoogt@uva.nl))

†† Nordic Institute for Product Sustainability, Environmental Chemistry and Toxicology (NIPSECT), 1 Dalgas Boulevard, DK-2000 Frederiksberg, Denmark ([allan.astrup.jensen@gmail.com](mailto:allan.astrup.jensen@gmail.com))

‡‡Wadsworth Center, New York State Department of Health, and Department of Environmental Health Sciences, School of Public Health, State University of New York at Albany, Empire State Plaza, P.O. Box 509, Albany, NY 12201-0509, USA ([kkannan@wadsworth.org](mailto:kkannan@wadsworth.org))

§§Department of Chemistry, University of Toronto, 80 St. George Street, Toronto, Ontario M5S 3H6, Canada ([smabury@chem.utoronto.ca](mailto:smabury@chem.utoronto.ca))

||||RIKILT - Institute of Food Safety, PO Box 230, 6700 AE Wageningen, The Netherlands ([Stefan.van.Leeuwen@WUR.nl](mailto:Stefan.van.Leeuwen@WUR.nl))

*To whom correspondence may be addressed: [james.franklin@skynet.be](mailto:james.franklin@skynet.be), Phone/Fax +32-10-24-69-98

Table of Contents

[PART 1 – FAMILIES OF SUBSTANCES (NON-POLYMERS) 4](#__RefHeading___Toc291669636)

[PART 2 – SELECTED INDIVIDUAL COMPOUNDS (NON-POLYMERS) 7](#__RefHeading___Toc291669637)

[Perfluoroalkyl carboxylic acids (and selected salts) 7](#__RefHeading___Toc291669638)

[Perfluoroalkyl carboxylate anions 8](#__RefHeading___Toc291669639)

[Perfluoroalkane sulfonic acids (and selected anions and salts) 10](#__RefHeading___Toc291669640)

[Perfluoroalkane sulfinic acids 11](#__RefHeading___Toc291669641)

[Perfluoroalkyl phosphonic acids 12](#__RefHeading___Toc291669642)

[Perfluoroalkyl phosphinic acids 12](#__RefHeading___Toc291669643)

[Perfluoroalkyl iodides 12](#__RefHeading___Toc291669644)

[(n:2) Fluorotelomer iodides 13](#__RefHeading___Toc291669645)

[(n:2) Fluorotelomer olefins 13](#__RefHeading___Toc291669646)

[(n:2) Fluorotelomer alcohols 14](#__RefHeading___Toc291669647)

[(n:2) Fluorotelomer acrylates 14](#__RefHeading___Toc291669648)

[(n:2) Fluorotelomer methacrylates 15](#__RefHeading___Toc291669649)

[Polyfluoroalkyl phosphoric acid monoesters 16](#__RefHeading___Toc291669650)

[Polyfluoroalkyl phosphoric acid diesters 16](#__RefHeading___Toc291669651)

[Semifluorinated n-alkanes 17](#__RefHeading___Toc291669652)

[Semifluorinated n-alkenes 17](#__RefHeading___Toc291669653)

[(n:2) Fluorotelomer (saturated) aldehydes 18](#__RefHeading___Toc291669654)

[(n:2) Fluorotelomer unsaturated aldehydes 18](#__RefHeading___Toc291669655)

[Perfluoroalkyl aldehydes 19](#__RefHeading___Toc291669656)

[Perfluoroalkyl aldehyde hydrates 19](#__RefHeading___Toc291669657)

[(n:2) Fluorotelomer (saturated) carboxylic acids 20](#__RefHeading___Toc291669658)

[(n:2) Fluorotelomer unsaturated carboxylic acids 20](#__RefHeading___Toc291669659)

[Other biotransformation products of n:2 FTOHs 21](#__RefHeading___Toc291669660)

[(n:2) Fluorotelomer sulfonic acids 21](#__RefHeading___Toc291669661)

[(n:2) Fluorotelomer sulfonate anions 22](#__RefHeading___Toc291669662)

[Perfluoroalkane sulfonyl fluorides 22](#__RefHeading___Toc291669663)

[Perfluoroalkane sulfonamides 23](#__RefHeading___Toc291669664)

[N-Methyl perfluoroalkane sulfonamides 23](#__RefHeading___Toc291669665)

[N-Ethyl perfluoroalkane sulfonamides 23](#__RefHeading___Toc291669666)

[N,N-Dialkyl perfluoroalkane sulfonamides 24](#__RefHeading___Toc291669667)

[Perfluoroalkane sulfonamido ethanols 24](#__RefHeading___Toc291669668)

[Perfluoroalkane sulfonamidoacetic acids 25](#__RefHeading___Toc291669669)

[N-Methyl perfluoroalkane sulfonamidoethanols 25](#__RefHeading___Toc291669670)

[N-Ethyl perfluoroalkane sulfonamidoethanols 26](#__RefHeading___Toc291669671)

[N-Methyl perfluoroalkane sulfonamidoacetic acids and salts 26](#__RefHeading___Toc291669672)

[N-Ethyl perfluoroalkane sulfonamidoacetic acids and salts 27](#__RefHeading___Toc291669673)

[N-Methyl perfluoroalkane sulfonamidoethyl acrylates 27](#__RefHeading___Toc291669674)

[N-Ethyl perfluoroalkane sulfonamidoethyl acrylates 28](#__RefHeading___Toc291669675)

[N-Methyl perfluoroalkane sulfonamidoethyl methacrylates 28](#__RefHeading___Toc291669676)

[N-Ethyl perfluoroalkane sulfonamidoethyl methacrylates 29](#__RefHeading___Toc291669677)

[Figure S1. Terminology Decision Flowcharts 30](#__RefHeading___Toc291669678)

[Example #1 8:2 Fluorotelomer alcohol 32](#__RefHeading___Toc291669679)

[Example #2 Perfluorobutane sulfonamide 33](#__RefHeading___Toc291669680)

[Example #3 Perfluorohexyl phosphonic acid 34](#__RefHeading___Toc291669681)

[Example #4 Side-chain Fluorinated Acrylate Polymer (Fluorotelomer origin) 35](#__RefHeading___Toc291669682)

# PART 1 – FAMILIES OF SUBSTANCES (NON-POLYMERS)

| **NAME OF FAMILY** | **FORMULA** | **ACRONYM** |
| --- | --- | --- |
| Perfluoroalkyl substances | Generic name: See main paper | PFASs |
| Perfluoroalkyl acids | Includes perfluoroalkyl carboxylic, sulfonic, sulfinic, phosphonic and phosphinic acids | PFAAs |
| Perfluoroalkyl carboxylic acids | C_n_F_2n+1_COOH | PFCAs |
| Perfluoroalkane sulfonic acids | C_n_F_2n+1_SO_3_H | PFSAs |
| Perfluoroalkane sulfinic acids | C_n_F_2n+1_SO_2_H | PFSIAs |
| Perfluoroalkyl phosphonic acids | O=P(OH)_2_C_n_F_2n+1_ | PFPAs |
| Perfluoroalkyl phosphinic acids | O=P(OH)(C_n_F_2n+1_)(C_m_F_2m+1_) | PFPIAs |
|  |  |  |
| **NAME OF FAMILY** | **FORMULA** | **ACRONYM** |
| Perfluoroalkyl iodides | C_n_F_2n+1_I | PFAIs |
| (n:2) Fluorotelomer iodides | C_n_F_2n+1_CH_2_CH_2_I | (n:2) FTIs |
| (n:2) Fluorotelomer olefins | C_n_F_2n+1_CH=CH_2_ | (n:2) FTOs |
| (n:2) Fluorotelomer alcohols | C_n_F_2n+1_CH_2_CH_2_OH | (n:2) FTOHs |
| (n:2) Fluorotelomer acrylates | C_n_F_2n+1_CH_2_CH_2_OC(O)CH=CH_2_ | (n:2) FTACs |
| (n:2) Fluorotelomer methacrylates | C_n_F_2n+1_CH_2_CH_2_OC(O)C(CH_3_)=CH_2_ | (n:2) FTMACs |

| Polyfluoroalkyl phosphoric acid esters / Polyfluoroalkyl phosphates / (n:2) Fluorotelomer phosphates | (O)P(OH)_3-x_(OCH_2_CH_2_C_n_F_2n+1_)_x_ | PAPs |
| --- | --- | --- |
| Polyfluoroalkyl phosphoric acid monoesters | (O)P(OH)_2_(OCH_2_CH_2_C_n_F_2n+1_) | monoPAPs |
| Polyfluoroalkyl phosphoric acid diesters | (O)P(OH)(OCH_2_CH_2_C_n_F_2n+1_)(OCH_2_CH_2_C_m_F_2m+1_) | diPAPs |
| Semi-fluorinated *n*-alkanes | F(CF_2_)_n_(CH_2_)_m_H | SFAs |
| Semi-fluorinated *n*-alkenes | F(CF_2_)_n_CH=CH(CH_2_)_m-2_H | SFAenes |
| (n:2) Fluorotelomer (saturated) aldehydes | C_n_F_2n+1_CH_2_CHO | (n:2) FTALs |
| (n:2) Fluorotelomer unsaturated aldehydes | C_n-1_F_2n-1_CF=CHCHO | (n:2) FTUALs |
| Perfluoroalkyl aldehydes | C_n_F_2n+1_CHO | PFALs |
| Perfluoroalkyl aldehyde hydrates | C_n_F_2n+1_CH(OH)_2_ | PFAL.H_2_Os |
| (n:2) Fluorotelomer (saturated) carboxylic acids | C_n_F_2n+1_CH_2_COOH | (n:2) FTCAs |
| (n:2) Fluorotelomer unsaturated carboxylic acids | C_n-1_F_2n-1_CF=CHCOOH | (n:2) FTUCAs |
| [Biotransformation product of (n+1):2 FTOH] | C_n_F_2n+1_CH_2_CH_2_COOH | n:3 Acid |
| [Biotransformation product of (n+1):2 FTOH] | C_n_F_2n+1_CH=CHCOOH | n:3 UAcid |
| (n:2) Fluorotelomer sulfonic acids | C_n_F_2n+1_CH_2_CH_2_SO_3_H | (n:2) FTSAs |

| **NAME OF FAMILY** | **FORMULA** | **ACRONYM** |
| --- | --- | --- |
| Perfluoroalkane sulfonyl fluorides | C_n_F_2n+1_SO_2_F | PASFs |
| Perfluoroalkane sulfonamides | C_n_F_2n+1_SO_2_NH_2_ | FASAs |
| *N*-Methyl perfluoroalkane sulfonamides | C_n_F_2n+1_SO_2_NH(CH_3_) | MeFASAs |
| *N*-Ethyl perfluoroalkane sulfonamides | C_n_F_2n+1_SO_2_NH(C_2_H_5_) | EtFASAs |
| *N*,*N*-Dialkyl perfluoroalkane sulfonamides | C_n_F_2n+1_SO_2_N(C_m_H_2m+1_)(C_p_H_2p+1_),  with m, p = 1 or 2 | Me_2_FASAs, Et_2_FASAs, MeEtFASAs |
| Perfluoroalkane sulfonamidoethanols | C_n_F_2n+1_SO_2_NHCH_2_CH_2_OH | FASEs |
| Perfluoroalkane sulfonamidoacetic acids | C_n_F_2n+1_SO_2_NHCH_2_COOH | FASAAs |
| *N*-Methyl perfluoroalkane sulfonamidoethanols | C_n_F_2n+1_SO_2_N(CH_3_)CH_2_CH_2_OH | MeFASEs |
| *N*-Ethyl perfluoroalkane sulfonamidoethanols | C_n_F_2n+1_SO_2_N(C_2_H_5_)CH_2_CH_2_OH | EtFASEs |
| *N*-Methyl perfluoroalkane sulfonamidoacetic acids | C_n_F_2n+1_SO_2_N(CH_3_)CH_2_COOH | MeFASAAs |
| *N*-Ethyl perfluoroalkane sulfonamidoacetic acids | C_n_F_2n+1_SO_2_N(C_2_H_5_)CH_2_COOH | EtFASAAs |
| *N*-Methyl perfluoroalkane sulfonamidoethyl acrylates | C_n_F_2n+1_SO_2_N(CH_3_)CH_2_CH_2_OC(O)CH=CH_2_ | MeFASACs |
| *N*-Ethyl perfluoroalkane sulfonamidoethyl acrylates | C_n_F_2n+1_SO_2_N(C_2_H_5_)CH_2_CH_2_OC(O)CH=CH_2_ | EtFASACs |
| *N*-Methyl perfluoroalkane sulfonamidoethyl methacrylates | C_n_F_2n+1_SO_2_N(CH_3_)CH_2_CH_2_OC(O)C(CH_3_)=CH_2_ | MeFASMACs |
| *N*-Ethyl perfluoroalkane sulfonamidoethyl methacrylates | C_n_F_2n+1_SO_2_N(C_2_H_5_)CH_2_CH_2_OC(O)C(CH_3_)=CH_2_ | EtFASMACs |

# PART 2 – SELECTED INDIVIDUAL COMPOUNDS (NON-POLYMERS)

| Perfluoroalkyl carboxylic acids (and selected salts) | **FORMULA** | **CAS REGISTRY NUMBER** | **ACRONYM** |
| --- | --- | --- | --- |
| Trifluoroacetic acid | CF_3_COOH | 76-05-1 | TFAA |
| Perfluoropropanoic acid | C_2_F_5_COOH | 422-64-0 | PFPrA |
| Perfluorobutanoic acid | C_3_F_7_COOH | 375-22-4 | PFBA |
| Ammonium perfluorobutanoate | NH_4_^+^ C_3_F_7_COO^-^ | 10495-86-0 | NH_4_-PFBA |
| Sodium perfluorobutanoate | Na^+^ C_3_F_7_COO^-^ | 2218-54-4 | Na-PFBA |
| Perfluoropentanoic acid | C_4_F_9_COOH | 2706-90-3 | PFPeA |
| Ammonium perfluoropentanoate | NH_4_^+^ C_4_F_9_COO^-^ | 68259-11-0 | NH_4_-PFPeA |
| Perfluorohexanoic acid | C_5_F_11_COOH | 307-24-4 | PFHxA |
| Ammonium perfluorohexanoate | NH_4_^+^ C_5_F_11_COO^-^ | 21615-47-4 | NH_4_-PFHxA |
| Sodium perfluorohexanoate | Na^+^ C_5_F_11_COO^-^ | 2923-26-4 | Na-PFHxA |
| Perfluoroheptanoic acid | C_6_F_13_COOH | 375-85-9 | PFHpA |
| Ammonium perfluoroheptanoate | NH_4_^+^ C_6_F_13_COO^-^ | 6130-43-4 | NH_4_-PFHpA |
| Sodium perfluoroheptanoate | Na^+^ C_6_F_13_COO^-^ | 20109-59-5 | Na-PFHpA |
| Perfluorooctanoic acid | C_7_F_15_COOH | 335-67-1 | PFOA |
| Ammonium perfluorooctanoate | NH_4_^+^ C_7_F_15_COO^-^ | 3825-26-1 | APFO (or  NH_4_-PFOA) |
| Sodium perfluorooctanoate | ²²²Na^+^ C_7_F_15_COO^-^ | 335-95-5 | Na-PFOA |
| Potassium perfluorooctanoate | K^+^ C_7_F_15_COO^-^ | 2395-00-8 | K-PFOA |
| Perfluorononanoic acid | C_8_F_17_COOH | 375-95-1 | PFNA |
| Ammonium perfluorononanoate | NH_4_^+^ C_8_F_17_COO^-^ | 4149-60-4 | APFN (or NH_4_-PFNA) |
| Sodium perfluorononanoate | Na^+^ C_8_F_17_COO^-^ | 21049-39-8 | Na-PFNA |
| Perfluorodecanoic acid | C_9_F_19_COOH | 335-76-2 | PFDA |
| Ammonium perfluorodecanoate | NH_4_^+^ C_9_F_19_COO^-^ | 3108-42-7 | NH_4_-PFDA |
| Perfluoroundecanoic acid | C_10_F_21_COOH | 2058-94-8 | PFUnDA |
| Ammonium perfluoroundecanoate | NH_4_^+^ C_10_F_21_COO^-^ | 4234-23-5 | NH_4_-PFUnDA |
| Perfluorododecanoic acid | C_11_F_23_COOH | 307-55-1 | PFDoDA |
| Perfluorotridecanoic acid | C_12_F_25_COOH | 72629-94-8 | PFTrDA |
| Perfluorotetradecanoic acid | C_13_F_27_COOH | 376-06-7 | PFTeDA |
| Perfluoropentadecanoic acid | C_14_F_29_COOH | 141074-63-7 | PFPeDA |
| Perfluorohexadecanoic acid | C_15_F_31_COOH | 67905-19-5 | PFHxDA |
| Perfluoroheptadecanoic acid | C_16_F_33_COOH | 57475-95-3 | PFHpDA |
| Perfluorooctadecanoic acid | C_17_F_35_COOH | 16517-11-6 | PFODA |
| Perfluoroalkyl carboxylate anions | **FORMULA** | **CAS REGISTRY NUMBER** | **ACRONYM** |
| Trifluoroacetate | CF_3_COO^-^ | 14477-72-6 | TFA |
| Perfluoropropanoate | C_2_F_5_COO^-^ | 44864-55-3 | PFPrA |
| Perfluorobutanoate | C_3_F_7_COO^-^ | 45048-62-2 | PFBA |
| Perfluoropentanoate | C_4_F_9_COO^-^ | 45167-47-3 | PFPeA |
| Perfluorohexanoate | C_5_F_11_COO^-^ | 92612-52-7 | PFHxA |
| Perfluorohepanoate | C_6_F_13_COO^-^ | 120885-29-2 | PFHpA |
| Perfluorooctanoate | C_7_F_15_COO^-^ | 45285-51-6 | PFOA |
| Perfluorononanoate | C_8_F_17_COO^-^ | 72007-68-2 | PFNA |
| Perfluorodecanoate | C_9_F_19_COO^-^ | 73829-36-4 | PFDA |
| Perfluoroundecanoate | C_10_F_21_COO^-^ | 196859-54-8 | PFUnDA |
| Perfluorododecanoate | C_11_F_23_COO^-^ | 171978-95-3 | PFDoDA |
| Perfluorotridecanoate | C_12_F_25_COO^-^ | 862374-87-6 | PFTrDA |
| Perfluorotetradecanoate | C_13_F_27_COO^-^ | 365971-87-5 | PFTeDA |
| Perfluoropentadecanoate | C_14_F_29_COO^-^ | 1214264-29-5 | PFPeDA |
| Perfluorohexadecanoate | C_15_F_31_COO^-^ | 1214264-30-8 | PFHxDA |
| Perfluoroheptadecanoate | C_16_F_33_COO^-^ | None available | PFHpDA |
| Perfluorooctadecanoate | C_17_F_35_COO^-^ | 798556-82-8 | PFODA |

| Perfluoroalkane sulfonic acids (and selected anions and salts) | **FORMULA** | **CAS REGISTRY NUMBER** | **ACRONYM** |
| --- | --- | --- | --- |
| Trifluoromethane sulfonic (or triflic) acid | CF_3_SO_3_H | 1493-13-6 | TFMS |
| Perfluoroethane sulfonic acid | C_2_F_5_SO_3_H | 354-88-1 | PFEtS |
| Perfluoropropane sulfonic acid | C_3_F_7_SO_3_H | 423-41-6 | PFPrS |
| Perfluorobutane sulfonic acid | C_4_F_9_SO_3_H | 375-73-5 or 59933-66-3 | PFBS |
| Perfluorobutane sulfonate anion | C_4_F_9_SO_3_^-^ | 45187-15-3 | PFBS |
| Potassium perfluorobutane sulfonate | K^+^ C_4_F_9_SO_3_^-^ | 29420-49-3 | K-PFBS |
| Perfluoropentane sulfonic acid | C_5_F_11_SO_3_H | 2706-91-4 | PFPeS |
| Potassium perfluopentane sulfonate | K^+^ C_5_F_11_SO_3_^-^ | 3872-25-1 | K-PFPeS |
| Perfluorohexane sulfonic acid | C_6_F_13_SO_3_H | 355-46-4 | PFHxS |
| Perfluorohexane sulfonate anion | C_6_F_13_SO_3_^-^ | 108427-53-8 | PFHxS |
| Potassium perfluorohexane sulfonate | K^+^ C_6_F_13_SO_3_^-^ | 3871-99-6 | K-PFHxS |
| Perfluoroheptane sulfonic acid | C_7_F_15_SO_3_H | 375-92-8 | PFHpS |
| Ammonium perfluoroheptane sulfonate | NH_4_^+^ C_7_F_15_SO_3_^-^ | 68259-07-4 | NH_4_-PFHpS |
| Potassium perfluoroheptane sulfonate | K^+^ C_7_F_15_SO_3_^-^ | 60270-55-5 | K-PFHpS |
| Perfluorooctane sulfonic acid | C_8_F_17_SO_3_H | 1763-23-1 | PFOS |
| Perfluorooctane sulfonate anion | C_8_F_17_SO_3_^-^ | 45298-90-6 | PFOS |
| Ammonium perfluorooctane sulfonate | NH_4_^+^ C_8_F_17_SO_3_^-^ | 29081-56-9 | NH_4_-PFOS |
| Sodium perfluorooctane sulfonate | Na^+^ C_8_F_17_SO_3_^-^ | 4021-47-0 | Na-PFOS |
| Potassium perfluorooctane sulfonate | K^+^ C_8_F_17_SO_3_^-^ | 2795-39-3 | K-PFOS |
| Lithium perfluorooctane sulfonate | Li^+^ C_8_F_17_SO_3_^-^ | 29457-72-5 | Li-PFOS |
| Tetraethylammonium perfluorooctane sulfonate | N(C_2_H_5_)_4_^+^ C_8_F_17_SO_3_^-^ | 56773-42-3 | NEt_4_-PFOS |
| Diethanolammonium perfluorooctane sulfonate | NH_2_(CH_2_CH_2_OH)_2_^+^ C_8_F_17_SO_3_^-^ | 56773-42-3 |  |
| Perfluorononane sulfonic acid | C_9_F_19_SO_3_H | 474511-07-4 | PFNS |
| Ammonium perfluorononane sulfonate | NH_4_^+^ C_9_F_19_SO_3_^-^ | 17202-41-4 | NH_4_-PFNS |
| Perfluorodecane sulfonic acid | C_10_F_21_SO_3_H | 335-77-3 | PFDS |
| Perfluorodecane sulfonate anion | C_10_F_21_SO_3_^-^ | 126105-34-8 | PFDS |
| Ammonium perfluorodecane sulfonate | NH_4_^+^ C_10_F_21_SO_3_^-^ | 67906-42-7 | NH_4_-PFDS |
| Potassium perfluorodecane sulfonate | K^+^ C_10_F_21_SO_3_^-^ | 2806-16-8 | K-PFDS |
| Perfluoroundecane sulfonic acid | C_11_F_23_SO_3_H | 749786-16-1 | PFUnDS |
| Perfluorododecane sulfonic acid | C_12_F_25_SO_3_H | 79780-39-5 | PFDoDS |
|  |  |  |  |
| Perfluoroalkane sulfinic acids | **FORMULA** | **CAS REGISTRY NUMBER** | **ACRONYM** |
| Perfluorooctane sulfinic acid | C_8_F_17_SO_2_H | 647-29-0 | PFOSI |

| Perfluoroalkyl phosphonic acids | **FORMULA** | **CAS REGISTRY NUMBER** | **ACRONYM** |
| --- | --- | --- | --- |
| Perfluorohexyl phosphonic acid | O=P(OH)_2_C_6_F_13_ | 40143-76-8 | C6-PFPA |
| Perfluorooctyl phosphonic acid | O=P(OH)_2_C_8_F_17_ | 40143-78-0 | C8-PFPA |
| Perfluorodecyl phosphonic acid | O=P(OH)_2_C_10_F_21_ | 52299-26-0 | C10-PFPA |
|  |  |  |  |
| Perfluoroalkyl phosphinic acids | **FORMULA** | **CAS REGISTRY NUMBER** | **ACRONYM** |
| Bis(perfluorohexyl) phosphinic acid | O=P(OH)(C_6_F_13_)_2_ | 40143-77-9 | C6/C6-PFPIA |
| Bis(perfluorooctyl) phosphinic acid | O=P(OH)(C_8_F_17_)_2_ | 40143-79-1 | C8/C8-PFPIA |
| Perfluoro(hexyloctyl) phosphinic acid | O=P(OH)(C_6_F_13_)(C_8_F_17_) | 610800-34-5 | C6/C8-PFPIA |
|  |  |  |  |
| Perfluoroalkyl iodides | **FORMULA** | **CAS REGISTRY NUMBER** | **ACRONYM** |
| Perfluoro (or pentafluoro)ethyl iodide | C_2_F_5_I | 354-64-3 | PFEI |
| Perfluorobutyl iodide | C_4_F_9_I | 423-39-2 | PFBI |
| Perfluorohexyl iodide | C_6_F_13_I | 355-43-1 | PFHxI |
| Perfluorooctyl iodide | C_8_F_17_I | 507-63-1 | PFOI |
| Perfluorodecyl iodide | C_10_F_21_I | 423-62-1 | PFDI |
| Perfluorododecyl iodide | C_12_F_25_I | 307-60-8 | PFDoDI |
| Perfluorotetradecyl iodide | C_14_F_29_I | 307-63-1 | PFTeDI |
| Perfluorohexadecyl iodide | C_16_F_33_I | 355-50-0 | PFHxDI |
| Perfluorooctadecyl iodide | C_18_F_37_I | 29809-35-6 | PFODI |
|  |  |  |  |
| (n:2) Fluorotelomer iodides | **FORMULA** | **CAS REGISTRY NUMBER** | **ACRONYM** |
| 4:2 Fluorotelomer iodide | C_4_F_9_CH_2_CH_2_I | 2043-55-2 | 4:2 FTI |
| 6:2 Fluorotelomer iodide | C_6_F_13_CH_2_CH_2_I | 2043-57-4 | 6:2 FTI |
| 8:2 Fluorotelomer iodide | C_8_F_17_CH_2_CH_2_I | 2043-53-0 | 8:2 FTI |
| 10:2 Fluorotelomer iodide | C_10_F_21_CH_2_CH_2_I | 2043-54-1 | 10:2 FTI |
| 12:2 Fluorotelomer iodide | C_12_F_25_CH_2_CH_2_I | 30046-31-2 | 12:2 FTI |
| 14:2 Fluorotelomer iodide | C_14_F_29_CH_2_CH_2_I | 65510-55-6 | 14:2 FTI |
| 16:2 Fluorotelomer iodide | C_16_F_33_CH_2_CH_2_I | 65150-94-9 | 16:2 FTI |
| 18:2 Fluorotelomer iodide | C_18_F_37_CH_2_CH_2_I | 65104-63-4 | 18:2 FTI |
|  |  |  |  |
| (n:2) Fluorotelomer olefins | **FORMULA** | **CAS REGISTRY NUMBER** | **ACRONYM** |
| 4:2 Fluorotelomer olefin | C_4_F_9_CH=CH_2_ | 19430-93-4 | 4:2 FTO |
| 6:2 Fluorotelomer olefin | C_6_F_13_CH=CH_2_ | 25291-17-2 | 6:2 FTO |
| 8:2 Fluorotelomer olefin | C_8_F_17_CH=CH_2_ | 21652-58-4 | 8:2 FTO |
| 10:2 Fluorotelomer olefin | C_10_F_21_CH=CH_2_ | 30389-25-4 | 10:2 FTO |
| 12:2 Fluorotelomer olefin | C_12_F_25_CH=CH_2_ | 67103-05-3 | 12:2 FTO |
|  |  |  |  |
| (n:2) Fluorotelomer alcohols | **FORMULA** | **CAS REGISTRY NUMBER** | **ACRONYM** |
| 4:2-Fluorotelomer alcohol | C_4_F_9_CH_2_CH_2_OH | 2043-47-2 | 4:2 FTOH |
| 6:2-Fluorotelomer alcohol | C_6_F_13_CH_2_CH_2_OH | 647-42-7 | 6:2 FTOH |
| 8:2-Fluorotelomer alcohol | C_8_F_17_CH_2_CH_2_OH | 678-39-7 | 8:2 FTOH |
| 10:2-Fluorotelomer alcohol | C_10_F_21_CH_2_CH_2_OH | 865-86-1 | 10:2 FTOH |
| 12:2 Fluorotelomer alcohol | C_12_F_25_CH_2_CH_2_OH | 39239-77-5 | 12:2 FTOH |
| 14:2 Fluorotelomer alcohol | C_14_F_29_CH_2_CH_2_OH | 60699-51-6 | 14:2 FTOH |
| 16:2 Fluorotelomer alcohol | C_16_F_33_CH_2_CH_2_OH | 65104-67-8 | 16:2 FTOH |
| 18:2 Fluorotelomer alcohol | C_18_F_37_CH_2_CH_2_OH | 65104-65-6 | 18:2 FTOH |
|  |  |  |  |
| (n:2) Fluorotelomer acrylates | **FORMULA** | **CAS REGISTRY NUMBER** | **ACRONYM** |
| 4:2 Fluorotelomer acrylate | C_4_F_9_CH_2_CH_2_OC(O)CH=CH_2_ | 52591-27-2 | 4:2 FTAC |
| 6:2 Fluorotelomer acrylate | C_6_F_13_CH_2_CH_2_OC(O)CH=CH_2_ | 17527-29-6 | 6:2 FTAC |
| 8:2 Fluorotelomer acrylate | C_8_F_17_CH_2_CH_2_OC(O)CH=CH_2_ | 27905-45-9 | 8:2 FTAC |
| 10:2 Fluorotelomer acrylate | C_10_F_21_CH_2_CH_2_OC(O)CH=CH_2_ | 17741-60-5 | 10:2 FTAC |
| 12:2 Fluorotelomer acrylate | C_12_F_25_CH_2_CH_2_OC(O)CH=CH_2_ | 34395-24-9 | 12:2 FTAC |
| 14:2 Fluorotelomer acrylate | C_14_F_29_CH_2_CH_2_OC(O)CH=CH_2_ | 34362-49-7 | 14:2 FTAC |
| 16:2 Fluorotelomer acrylate | C_16_F_33_CH_2_CH_2_OC(O)CH=CH_2_ | 65150-93-8 | 16:2 FTAC |
| 18:2 Fluorotelomer acrylate | C_18_F_37_CH_2_CH_2_OC(O)CH=CH_2_ | 65104-64-5 | 18:2 FTAC |
|  |  |  |  |
| (n:2) Fluorotelomer methacrylates | **FORMULA** | **CAS REGISTRY NUMBER** | **ACRONYM** |
| 4:2 Fluorotelomer methacrylate | C_4_F_9_CH_2_CH_2_OC(O)C(CH_3_)=CH_2_ | 1799-84-4 | 4:2 FTMAC |
| 6:2 Fluorotelomer methacrylate | C_6_F_13_CH_2_CH_2_OC(O)C(CH_3_)=CH_2_ | 2144-53-8 | 6:2 FTMAC |
| 8:2 Fluorotelomer methacrylate | C_8_F_17_CH_2_CH_2_OC(O)C(CH_3_)=CH_2_ | 1996-88-9 | 8:2 FTMAC |
| 10:2 Fluorotelomer methacrylate | C_10_F_21_CH_2_CH_2_OC(O)C(CH_3_)=CH_2_ | 2144-54-9 | 10:2 FTMAC |
| 12:2 Fluorotelomer methacrylate | C_12_F_25_CH_2_CH_2_OC(O)C(CH_3_)=CH_2_ | 6014-75-1 | 12:2 FTMAC |
| 14:2 Fluorotelomer methacrylate | C_14_F_29_CH_2_CH_2_OC(O)C(CH_3_)=CH_2_ | 4980-53-4 | 14:2 FTMAC |
| 16:2 Fluorotelomer methacrylate | C_16_F_33_CH_2_CH_2_OC(O)C(CH_3_)=CH_2_ | 59778-97-1 | 16:2 FTMAC |
| 18:2 Fluorotelomer methacrylate | C_18_F_37_CH_2_CH_2_OC(O)C(CH_3_)=CH_2_ | 65104-66-7 | 18:2 FTMAC |

| Polyfluoroalkyl phosphoric acid monoesters **(= fluorotelomer phosphate monoesters)** | **FORMULA** | **CAS REGISTRY NUMBER** | **ACRONYM** |
| --- | --- | --- | --- |
| 4:2 Fluorotelomer phosphate monoester | (O)P(OH)_2_(OCH_2_CH_2_C_4_F_9_) | 150065-76-2 | 4:2 monoPAP |
| 6:2 Fluorotelomer phosphate monoester | (O)P(OH)_2_(OCH_2_CH_2_C_6_F_13_) | 57678-01-0 | 6:2 monoPAP |
| 8:2 Fluorotelomer phosphate monoester | (O)P(OH)_2_(OCH_2_CH_2_C_8_F_17_) | 57678-03-2 | 8:2 monoPAP |
| 10:2 Fluorotelomer phosphate monoester | (O)P(OH)_2_(OCH_2_CH_2_C_10_F_21_) | 57678-05-4 | 10:2 monoPAP |
| 12:2 Fluorotelomer phosphate monoester | (O)P(OH)_2_(OCH_2_CH_2_C_12_F_25_) | 57678-07-6 | 12:2 monoPAP |
| Polyfluoroalkyl phosphoric acid diesters  **(= fluorotelomer phosphate diesters)** | **FORMULA** | **CAS REGISTRY NUMBER** | **ACRONYM** |
| 4:2 Fluorotelomer phosphate diester | (O)P(OH)(OCH_2_CH_2_C_4_F_9_)_2_ | 135098-69-0 | 4:2 diPAP |
| 4:2/6:2 Fluorotelomer phosphate diester | (O)P(OH)(OCH_2_CH_2_C_4_F_9_)(OCH_2_CH_2_C_6_F_13_) | 1158182-59-2 | 4:2/6:2 diPAP |
| 6:2 Fluorotelomer phosphate diester | (O)P(OH)(OCH_2_CH_2_C_6_F_13_)_2_ | 57677-95-9 | 6:2 diPAP |
| 6:2/8:2 Fluorotelomer phosphate diester | (O)P(OH)(OCH_2_CH_2_C_6_F_13_)(OCH_2_CH_2_C_8_F_17_) | 943913-15-3 | 6:2/8:2 diPAP |
| 8:2 Fluorotelomer phosphate diester | (O)P(OH)(OCH_2_CH_2_C_8_F_17_)_2_ | 678-41-1 | 8:2 diPAP |
| 8:2/10:2 Fluorotelomer phosphate diester | (O)P(OH)(OCH_2_CH_2_C_8_F_17_)(OCH_2_CH_2_C_10_F_21_) | 1158182-60-5 | 8:2/10:2 diPAP |
| 10:2 Fluorotelomer phosphate diester | (O)P(OH)(OCH_2_CH_2_C_10_F_21_)_2_ | 1895-26-7 | 10:2 diPAP |
| 10:2/12:2 Fluorotel. phosphate diester | (O)P(OH)(OCH_2_CH_2_C_10_F_21_)(OCH_2_CH_2_C_12_F_25_) | 1158182-61-6 | 10:2/12:2 diPAP |
| 12:2 Fluorotelomer phosphate diester | (O)P(OH)(OCH_2_CH_2_C_12_F_25_)_2_ | 57677-99-3 | 12:2 diPAP |
| Semifluorinated n-alkanes | **FORMULA** | **CAS REGISTRY NUMBER** | **ACRONYM** |
| (Perfluorooctyl)ethane | F(CF_2_)_8_(CH_2_)_2_H | 77117-48-7 | F_8_H_2_ |
| (Perfluorohexyl)octane | F(CF_2_)_6_(CH_2_)_8_H | 133331-77-8 | F_6_H_8_ |
| (Perfluorohexyl)hexadecane | F(CF_2_)_6_(CH_2_)_16_H | 133310-71-1 | F_6_H_16_ |
| (Perfluorooctyl)hexadecane | F(CF_2_)_8_(CH_2_)_16_H | 117146-18-6 | F_8_H_16_ |
| (Perfluorohexadecyl)hexadecane | F(CF_2_)_16_(CH_2_)_16_H | 137338-42-2 | F_16_H_16_ |
|  |  |  |  |
| Semifluorinated n-alkenes | **FORMULA** | **CAS REGISTRY NUMBER** | **ACRONYM** |
| (Perfluorohexyl)hexadecene | F(CF_2_)_6_CH=CH(CH_2_)_14_H | 1244062-15-4 | F_6_H_16_ene |
| (Perfluorooctyl)hexadecene | F(CF_2_)_8_CH=CH(CH_2_)_14_H | 1244062-16-5 | F_8_H_16_ene |
| (Perfluorohexadecyl)hexadecene | F(CF_2_)_16_CH=CH(CH_2_)_14_H | 1244062-14-3 | F_16_H_16_ene |

| (n:2) Fluorotelomer (saturated) aldehydes | **FORMULA** | **CAS REGISTRY NUMBER** | **ACRONYM** |
| --- | --- | --- | --- |
| 4:2 Fluorotelomer aldehyde | C_4_F_9_CH_2_CHO | 135984-67-7 | 4:2 FTAL |
| 6:2 Fluorotelomer aldehyde | C_6_F_13_CH_2_CHO | 56734-81-7 | 6:2 FTAL |
| 8:2 Fluorotelomer aldehyde | C_8_F_17_CH_2_CHO | 135984-68-8 | 8:2 FTAL |
| 10:2 Fluorotelomer aldehyde | C_10_F_21_CH_2_CHO | 864551-38-2 | 10:2 FTAL |
| 12:2 Fluorotelomer aldehyde | C_12_F_25_CH_2_CHO | None available | 12:2 FTAL |
|  |  |  |  |
| (n:2) Fluorotelomer unsaturated aldehydes | **FORMULA** | **CAS REGISTRY NUMBER** | **ACRONYM** |
| 4:2 Fluorotelomer unsaturated aldehyde | C_3_F_7_CF=CHCHO | 864551-39-3 | 4:2 FTUAL |
| 6:2 Fluorotelomer unsaturated aldehyde | C_5_F_11_CF=CHCHO | 69534-12-9 | 6:2 FTUAL |
| 8:2 Fluorotelomer unsaturated aldehyde | C_7_F_15_CF=CHCHO | 58544-13-1 | 8:2 FTUAL |
| 10:2 Fluorotelomer unsaturated aldehyde | C_9_F_19_CF=CHCHO | 864551-40-6 | 10:2 FTUAL |
| 12:2 Fluorotelomer unsaturated aldehyde | C_11_F_23_CF=CHCHO | None available | 12:2 FTUAL |

| Perfluoroalkyl aldehydes | **FORMULA** | **CAS REGISTRY NUMBER** | **ACRONYM** |
| --- | --- | --- | --- |
| Perfluoropentanal | C_4_F_9_CHO | 375-53-1 | PFPeAL |
| Perfluoroheptanal | C_6_F_13_CHO | 63967-41-9 | PFHpAL |
| Perfluorooctanal | C_7_F_15_CHO | 335-60-4 | PFOAL |
| Perfluorononanal | C_8_F_17_CHO | 63967-40-8 | PFNAL |
| Perfluoroundecanal | C_10_F_21_CHO | 63967-42-0 | PFUnDAL |
|  |  |  |  |
| Perfluoroalkyl aldehyde hydrates | **FORMULA** | **CAS REGISTRY NUMBER** | **ACRONYM** |
| Perfluoropentanal hydrate | C_4_F_9_CH(OH)_2_ | 355-30-6 | PFPeAL.H_2_O |
| Perfluoroheptanal hydrate | C_6_F_13_CH(OH)_2_ | 64739-16-8 | PFHpAL.H_2_O |
| Perfluorononanal hydrate | C_8_F_17_CH(OH)_2_ | 191528-99-1 | PFNAL.H_2_O |
| Perfluoroundecanal hydrate | C_10_F_21_CH(OH)_2_ | None Available | PFUnDAL.H_2_O |

| (n:2) Fluorotelomer (saturated) carboxylic acids | **FORMULA** | **CAS REGISTRY NUMBER** | **ACRONYM** |
| --- | --- | --- | --- |
| 6:2 Fluorotelomer carboxylic acid | C_6_F_13_CH_2_COOH | 53826-12-3 | 6:2 FTCA |
| 8:2 Fluorotelomer carboxylic acid | C_8_F_17_CH_2_COOH | 27854-31-5 | 8:2 FTCA |
| 10:2 Fluorotelomer carboxylic acid | C_10_F_21_CH_2_COOH | 53826-13-4 | 10:2 FTCA |
| 12:2 Fluorotelomer carboxylic acid | C_12_F_25_CH_2_COOH | 70887-93-3 | 12:2 FTCA |
|  |  |  |  |
| (n:2) Fluorotelomer unsaturated carboxylic acids | **FORMULA** | **CAS REGISTRY NUMBER** | **ACRONYM** |
| 6:2 Fluorotelomer unsaturated carboxylic acid | C_5_F_11_CF=CHCOOH | 70887-88-6 | 6:2 FTUCA |
| 8:2 Fluorotelomer unsaturated carboxylic acid | C_7_F_15_CF=CHCOOH | 70887-84-2 | 8:2 FTUCA |
| 10:2 Fluorotelomer unsaturated carboxylic acid | C_9_F_19_CF=CHCOOH | 70887-94-4 | 10:2 FTUCA |
| 12:2 Fluorotelomer unsaturated carboxylic acid | C_11_F_23_CF=CHCOOH | 70887-95-5 | 12:2 FTUCA |

| Other biotransformation products of n:2 FTOHs | **FORMULA** | **CAS REGISTRY NUMBER** | **ACRONYM** |
| --- | --- | --- | --- |
| 4:3 Acid | C_4_F_9_(CH_2_)_2_COOH | 80705-13-1 | 4:3 Acid |
| 5:3 Acid | C_5_F_11_(CH_2_)_2_COOH | 914637-49-3 | 5:3 Acid |
| 6:3 Acid | C_6_F_13_(CH_2_)_2_COOH | 27854-30-4 | 6:3 Acid |
| 7:3 Acid | C_7_F_15_(CH_2_)_2_COOH | 812-70-4 | 7:3 Acid |
| 5:3 Unsaturated carboxylic acid | C_5_F_11_CH=CHCOOH | 1869-04-1  875878-70-9 (E) | 5:3 UAcid |
| 7:3 Unsaturated carboxylic acid | C_7_F_15_CH=CHCOOH | 755-03-3  56017-63-1 (E)  173441-56-0 (Z) | 7:3 UAcid |
|  |  |  |  |
| (n:2) Fluorotelomer sulfonic acids | **FORMULA** | **CAS REGISTRY NUMBER** | **ACRONYM** |
| 4:2 Fluorotelomer sulfonic acid | C_4_F_9_CH_2_CH_2_SO_3_H | 757124-72-4 | 4:2 FTSA |
| 6:2 Fluorotelomer sulfonic acid | C_6_F_13_CH_2_CH_2_SO_3_H | 27619-97-2 | 6:2 FTSA |
| 8:2 Fluorotelomer sulfonic acid | C_8_F_17_CH_2_CH_2_SO_3_H | 39108-34-4 | 8:2 FTSA |
| 10:2 Fluorotelomer sulfonic acid | C_10_F_21_CH_2_CH_2_SO_3_H | 120226-60-0 | 10:2 FTSA |

| (n:2) Fluorotelomer sulfonate anions | **FORMULA** | **CAS REGISTRY NUMBER** | **ACRONYM** |
| --- | --- | --- | --- |
| 4:2 Fluorotelomer sulfonate anion | C_4_F_9_CH_2_CH_2_SO_3_^-^ | 414911-30-1 | 4:2 FTSA |
| 6:2 Fluorotelomer sulfonate anion | C_6_F_13_CH_2_CH_2_SO_3_^-^ | 425670-75-3 | 6:2 FTSA |
| 8:2 Fluorotelomer sulfonate anion | C_8_F_17_CH_2_CH_2_SO_3_^-^ | 481071-78-7 | 8:2 FTSA |
| 10:2 Fluorotelomer sulfonate anion | C_10_F_21_CH_2_CH_2_SO_3_^-^ | None available | 10:2 FTSA |
|  |  |  |  |
| Perfluoroalkane sulfonyl fluorides | **FORMULA** | **CAS REGISTRY NUMBER** | **ACRONYM** |
| Perfluorobutane sulfonyl fluoride | C_4_F_9_SO_2_F | 375-72-4 | PBSF |
| Perfluoropentane sulfonyl fluoride | C_5_F_11_SO_2_F | 375-81-5 | PPeSF |
| Perfluorohexane sulfonyl fluoride | C_6_F_13_SO_2_F | 423-50-7 | PHxSF |
| Perfluoroheptane sulfonyl fluoride | C_7_F_15_SO_2_F | 335-71-7 | PHpSF |
| Perfluorooctane sulfonyl fluoride | C_8_F_17_SO_2_F | 307-35-7 | POSF |
| Perfluorononane sulfonyl fluoride | C_9_F_19_SO_2_F | 68259-06-3 | PNSF |
| Perfluorodecane sulfonyl fluoride | C_10_F_21_SO_2_F | 307-51-7 | PDSF |

| Perfluoroalkane sulfonamides | **FORMULA** | **CAS REGISTRY NUMBER** | **ACRONYM** |
| --- | --- | --- | --- |
| Perfluorobutane sulfonamide | C_4_F_9_SO_2_NH_2_ | 30334-69-1 | FBSA |
| Perfluoropentane sulfonamide | C_5_F_11_SO_2_NH_2_ | 82765-76-2 | FPeSA |
| Perfluorohexane sulfonamide | C_6_F_13_SO_2_NH_2_ | 41997-13-1 | FHxSA |
| Perfluoroheptane sulfonamide | C_7_F_15_SO_2_NH_2_ | 82765-77-3 | FHpSA |
| Perfluorooctane sulfonamide | C_8_F_17_SO_2_NH_2_ | 754-91-6 | FOSA |
| N-Methyl perfluoroalkane sulfonamides | **FORMULA** | **CAS REGISTRY NUMBER** | **ACRONYM** |
| *N*-Methyl perfluorobutane sulfonamide | C_4_F_9_SO_2_NH(CH_3_) | 68298-12-4 | MeFBSA |
| *N*-Methyl perfluoropentane sulfonamide | C_5_F_11_SO_2_NH(CH_3_) | 68298-13-5 | MeFPeSA |
| *N*-Methyl perfluorohexane sulfonamide | C_6_F_13_SO_2_NH(CH_3_) | 68259-15-4 | MeFHxSA |
| *N*-Methyl perfluoroheptane sulfonamide | C_7_F_15_SO_2_NH(CH_3_) | 68259-14-3 | MeFHpSA |
| *N*-Methyl perfluorooctane sulfonamide | C_8_F_17_SO_2_NH(CH_3_) | 31506-32-8 | MeFOSA |
| N-Ethyl perfluoroalkane sulfonamides | **FORMULA** | **CAS REGISTRY NUMBER** | **ACRONYM** |
| *N*-Ethyl perfluorobutane sulfonamide | C_4_F_9_SO_2_NH(C_2_H_5_) | 40630-67-9 | EtFBSA |
| *N*-Ethyl perfluoropentane sulfonamide | C_5_F_11_SO_2_NH(C_2_H_5_) | 162682-16-8 | EtFPeSA |
| *N*-Ethyl perfluorohexane sulfonamide | C_6_F_13_SO_2_NH(C_2_H_5_) | 87988-56-5 | EtFHxSA |
| *N*-Ethyl perfluoroheptane sulfonamide | C_7_F_15_SO_2_NH(C_2_H_5_) | 68957-62-0 | EtFHpSA |
| *N*-Ethyl perfluorooctane sulfonamide | C_8_F_17_SO_2_NH(C_2_H_5_) (sulfluramid) | 4151-50-2 | EtFOSA |
| N,N-Dialkyl perfluoroalkane sulfonamides | **FORMULA** | **CAS REGISTRY NUMBER** | **ACRONYM** |
| *N,N*-Dimethyl perfluorooctane sulfonamide | C_8_F_17_SO_2_N(CH_3_)_2_ | 213181-78-3 | Me_2_FOSA |
| *N,N*-Diethyl perfluorooctane sulfonamide | C_8_F_17_SO_2_N(C_2_H_5_)_2_ | 87988-61-2 | Et_2_FOSA |
|  |  |  |  |
| Perfluoroalkane sulfonamido ethanols | **FORMULA** | **CAS REGISTRY NUMBER** | **ACRONYM** |
| Perfluorobutane sulfonamidoethanol | C_4_F_9_SO_2_NHCH_2_CH_2_OH | 34454-99-4 | FBSE |
| Perfluoropentane sulfonamidoethanol | C_5_F_11_SO_2_NHCH_2_CH_2_OH | None available | FPeSE |
| Perfluorohexane sulfonamidoethanol | C_6_F_13_SO_2_NHCH_2_CH_2_OH | 106443-63-4 | FHxSE |
| Perfluoroheptane sulfonamidoethanol | C_7_F_15_SO_2_NHCH_2_CH_2_OH | 167398-54-1 | FHpSE |
| Perfluorooctane sulfonamidoethanol | C_8_F_17_SO_2_NHCH_2_CH_2_OH | 10116-92-4 | FOSE |

| Perfluoroalkane sulfonamidoacetic acids | **FORMULA** | **CAS REGISTRY NUMBER** | **ACRONYM** |
| --- | --- | --- | --- |
| Perfluorobutane sulfonamidoacetic acid | C_4_F_9_SO_2_NHCH_2_COOH | 347872-22-4 | FBSAA |
| Perfluoropentane sulfonamidoacetic acid | C_5_F_11_SO_2_NHCH_2_COOH | 647-43-8 | FPeSAA |
| Perfluorohexane sulfonamidoacetic acid | C_6_F_2n+1_SO_2_NHCH_2_COOH | 1003193-99-4 | FHxSAA |
| Perfluoroheptane sulfonamidoacetic acid | C_7_F_15_SO_2_NHCH_2_COOH | 1003194-00-0 | FHpSAA |
| Perfluorooctane sulfonamidoacetic acid | C_8_F_17_SO_2_NHCH_2_COOH | 2806-24-8 | FOSAA |
|  |  |  |  |
| N-Methyl perfluoroalkane sulfonamidoethanols | **FORMULA** | **CAS REGISTRY NUMBER** | **ACRONYM** |
| *N*-Methyl perfluorobutane sulfonamidoethanol | C_4_F_9_SO_2_N(CH_3_)CH_2_CH_2_OH | 34454-97-2 | MeFBSE |
| *N*-Methyl perfluoropentane sulfonamidoethanol | C_5_F_11_SO_2_N(CH_3_)CH_2_CH_2_OH | 68555-74-8 | MeFPeSE |
| *N*-Methyl perfluorohexane sulfonamidoethanol | C_6_F_13_SO_2_N(CH_3_)CH_2_CH_2_OH | 68555-75-9 | MeFHxSE |
| *N*-Methyl perfluoroheptane sulfonamidoethanol | C_7_F_15_SO_2_N(CH_3_)CH_2_CH_2_OH | 68555-76-0 | MeFHpSE |
| *N*-Methyl perfluorooctane sulfonamidoethanol | C_8_F_17_SO_2_N(CH_3_)CH_2_CH_2_OH | 24448-09-7 | MeFOSE |
| N-Ethyl perfluoroalkane sulfonamidoethanols | **FORMULA** | **CAS REGISTRY NUMBER** | **ACRONYM** |
| *N*-Ethyl perfluorobutane sulfonamidoethanol | C_4_F_9_SO_2_N(C_2_H_5_)CH_2_CH_2_OH | 34449-89-3 | EtFBSE |
| *N*-Ethyl perfluoropentane sulfonamidoethanol | C_5_F_11_SO_2_N(C_2_H_5_)CH_2_CH_2_OH | 68555-72-6 | EtFPeSE |
| *N*-Ethyl perfluorohexane sulfonamidoethanol | C_6_F_13_SO_2_N(C_2_H_5_)CH_2_CH_2_OH | 34455-03-3 | EtFHxSE |
| *N*-Ethyl perfluoroheptane sulfonamidoethanol | C_7_F_15_SO_2_N(C_2_H_5_)CH_2_CH_2_OH | 68555-73-7 | EtFHpSE |
| *N*-Ethyl perfluorooctane sulfonamidoethanol | C_8_F_17_SO_2_N(C_2_H_5_)CH_2_CH_2_OH | 1691-99-2 | EtFOSE |
|  |  |  |  |
| N-Methyl perfluoroalkane sulfonamidoacetic acids and salts | **FORMULA** | **CAS REGISTRY NUMBER** | **ACRONYM** |
| *N*-Methyl perfluorobutane sulfonamidoacetic acid | C_4_F_9_SO_2_N(CH_3_)CH_2_COOH | 159381-10-9 | MeFBSAA |
| *N*-Methyl perfluorohexane sulfonamidoacetic acid | C_6_F_13_SO_2_N(CH_3_)CH_2_COOH | 715646-50-7 | MeFHxSAA |
| *N*-Methyl perfluorooctane sulfonamidoacetic acid | C_8_F_17_SO_2_N(CH_3_)CH_2_COOH | 2355-31-9 | MeFOSAA |
| Potassium *N*-methyl perfluorooctane sulfonamidoacetate | K^+^ C_8_F_17_SO_2_N(CH_3_)CH_2_COO^-^ | 70281-93-5 | K-MeFOSAA |
| N-Ethyl perfluoroalkane sulfonamidoacetic acids and salts | **FORMULA** | **CAS REGISTRY NUMBER** | **ACRONYM** |
| *N*-Ethyl perfluorobutane sulfonamidoacetic acid | C_4_F_9_SO_2_N(C_2_H_5_)CH_2_COOH | 68957-33-5 | EtFBSAA |
| *N*-Ethyl perfluoropentane sulfonamidoacetic acid | C_5_F_11_SO_2_N(C_2_H_5_)CH_2_COOH | 68957-31-3 | EtFPeSAA |
| *N*-Ethyl perfluorohexane sulfonamidoacetic acid | C_6_F_13_SO_2_N(C_2_H_5_)CH_2_COOH | 68957-32-4 | EtFHxSAA |
| *N*-Ethyl perfluoroheptane sulfonamidoacetic acid | C_7_F_15_SO_2_N(C_2_H_5_)CH_2_COOH | 68957-63-1 | EtFHpSAA |
| *N*-Ethyl perfluorooctane sulfonamidoacetic acid | C_8_F_17_SO_2_N(C_2_H_5_)CH_2_COOH | 2991-50-6 | EtFOSAA |
| Potassium *N*-ethyl perfluorooctane sulfonamidoacetate | K^+^ C_8_F_17_SO_2_N(C_2_H_5_)CH_2_COO^-^ | 2991-51-7 | K-EtFOSAA |
|  |  |  |  |
| N-Methyl perfluoroalkane sulfonamidoethyl acrylates | **FORMULA** | **CAS REGISTRY NUMBER** | **ACRONYM** |
| *N*-Methyl perfluorobutane sulfonamidoethyl acrylate | C_4_F_9_SO_2_N(CH_3_)CH_2_CH_2_OC(O)CH=CH_2_ | 67584-55-8 | MeFBSAC |
| *N*-Methyl perfluoropentane sulfonamidoethyl acrylate | C_5_F_11_SO_2_N(CH_3_)CH_2_CH_2_OC(O)CH=CH_2_ | 67584-56-9 | MeFPeSAC |
| *N*-Methyl perfluorohexane sulfonamidoethyl acrylate | C_6_F_13_SO_2_N(CH_3_)CH_2_CH_2_OC(O)CH=CH_2_ | 67584-57-0 | MeFHxSAC |
| *N*-Methyl perfluoroheptane sulfonamidoethyl acrylate | C_7_F_15_SO_2_N(CH_3_)CH_2_CH_2_OC(O)CH=CH_2_ | 68084-62-8 | MeFHpSAC |
| *N*-Methyl perfluorooctane sulfonamidoethyl acrylate | C_8_F_17_SO_2_N(CH_3_)CH_2_CH_2_OC(O)CH=CH_2_ | 25268-77-3 | MeFOSAC |
| N-Ethyl perfluoroalkane sulfonamidoethyl acrylates | **FORMULA** | **CAS REGISTRY NUMBER** | **ACRONYM** |
| *N*-Ethyl perfluorobutane sulfonamidoethyl acrylate | C_4_F_9_SO_2_N(C_2_H_5_)CH_2_CH_2_OC(O)CH=CH_2_ | 17329-79-2 | EtFBSAC |
| *N*-Ethyl perfluoropentane sulfonamidoethyl acrylate | C_5_F_11_SO_2_N(C_2_H_5_)CH_2_CH_2_OC(O)CH=CH_2_ | 68298-06-6 | EtFPeSAC |
| *N*-Ethyl perfluorohexane sulfonamidoethyl acrylate | C_6_F_13_SO_2_N(C_2_H_5_)CH_2_CH_2_OC(O)CH=CH_2_ | 1893-52-3 | EtFHxSAC |
| *N*-Ethyl perfluoroheptane sulfonamidoethyl acrylate | C_7_F_15_SO_2_N(C_2_H_5_)CH_2_CH_2_OC(O)CH=CH_2_ | 59071-10-2 | EtFHpSAC |
| *N*-Ethyl perfluorooctane sulfonamidoethyl acrylate | C_8_F_17_SO_2_N(C_2_H_5_)CH_2_CH_2_OC(O)CH=CH_2_ | 423-82-5 | EtFOSAC |
| N-Methyl perfluoroalkane sulfonamidoethyl methacrylates | **FORMULA** | **CAS REGISTRY NUMBER** | **ACRONYM** |
| *N*-Methyl perfluorobutane sulfonamidoethyl methacrylate | C_4_F_9_SO_2_N(CH_3_)CH_2_CH_2_OC(O)C(CH_3_)=CH_2_ | 67584-59-2 | MeFBSMAC |
| *N*-Methyl perfluoropentane sulfonamidoethyl methacrylate | C_5_F_11_SO_2_N(CH_3_)CH_2_CH_2_OC(O)C(CH_3_)=CH_2_ | 67584-60-5 | MeFPeSMAC |
| *N*-Methyl perfluorohexane sulfonamidoethyl methacrylate | C_6_F_13_SO_2_N(CH_3_)CH_2_CH_2_OC(O)C(CH_3_)=CH_2_ | 67584-61-6 | MeFHxSMAC |
| *N*-Methyl perfluoroheptane sulfonamidoethyl methacrylate | C_7_F_15_SO_2_N(CH_3_)CH_2_CH_2_OC(O)C(CH_3_)=CH_2_ | 67939-96-2 | MeFHpSMAC |
| *N*-Methyl perfluorooctane sulfonamidoethyl methacrylate | C_8_F_17_SO_2_N(CH_3_)CH_2_CH_2_OC(O)C(CH_3_)=CH_2_ | 14650-24-9 | MeFOSMAC |
| N-Ethyl perfluoroalkane sulfonamidoethyl methacrylates | **FORMULA** | **CAS REGISTRY NUMBER** | **ACRONYM** |
| *N*-Ethyl perfluorobutane sulfonamidoethyl methacrylate | C_4_F_9_SO_2_N(C_2_H_5_)CH_2_CH_2_OC(O)C(CH_3_)=CH_2_ | 67939-33-7 | EtFBSMAC |
| *N*-Ethyl perfluoropentane sulfonamidoethyl methacrylate | C_5_F_11_SO_2_N(C_2_H_5_)CH_2_CH_2_OC(O)C(CH_3_)=CH_2_ | 67906-73-4 | EtFPeSMAC |
| *N*-Ethyl perfluorohexane sulfonamidoethyl methacrylate | C_6_F_13_SO_2_N(C_2_H_5_)CH_2_CH_2_OC(O)C(CH_3_)=CH_2_ | 67906-70-1 | EtFHxSMAC |
| *N*-Ethyl perfluoroheptane sulfonamidoethyl methacrylate | C_7_F_15_SO_2_N(C_2_H_5_)CH_2_CH_2_OC(O)C(CH_3_)=CH_2_ | 67939-36-0 | EtFHpSMAC |
| *N*-Ethyl perfluorooctane sulfonamidoethyl methacrylate | C_8_F_17_SO_2_N(C_2_H_5_)CH_2_CH_2_OC(O)C(CH_3_)=CH_2_ | 376-14-7 | EtFOSMAC |

# Figure S1. Terminology Decision Flowcharts

**Fluorinated Polymer Decision**

**Non-Polymer Decision Tree**

**Non-**

**Polymer**

**Is the**

**substance**

**perfluorinated**

**?**

**Poly**

**fluorinated**

**Substance**

**Per**

**fluorinated**

**Substance**

What is the functional group

“

X

”

attached to the

perfluoroalkyl

chain in

C

n

F

2n+1

X ?

What

’

s

“

X?

”

1.

What is the atomic

composition and

chemical structure of

the polyfluorinated

substance?

2.

What commercial

manufacturing process

does the substance

originate from?

**X = F**

**Perfluorocarbon**

**X = C, P, or S oxidized**

**Perfluoroalkyl Acid**

**X = I,**

**Cl**

**, Br**

**Perfluoroalkyl Halide**

**X = CHO**

**Perfluoroalkyl**

**Aldehyde**

**Fluorotelomer Based**

**Electrochemical**

**Fluorination (ECF)**

**Based**

**Fluorinated**

**(Poly) Ether Based**

## Example #1 8:2 Fluorotelomer alcohol

| **Substance Chemical Structure** | **Question** | **Conclusion** |
| --- | --- | --- |
| **F(CF_2_)_8_CH_2_CH_2_OH** | **Polymer or Non-Polymer?** | **Non-Polymer** |
| **F(CF_2_)_8_**CH_2_CH_2_OH | **Perfluorinated?**  *No. The substance has a perfluoroalkyl chain, F(CF_2_)_8_-, but all hydrogen on carbons are not replaced with fluorine* | **Poly-fluorinated** |
| F(CF_2_)_8_**CH_2_CH_2_**OH | **Process Origin?**  *Perfluoroalkyl chain with an ethylene spacer (-CH_2_CH_2_-). Fluorotelomer origin* | **Fluoro-telomer origin** |
| F(CF_2_)_8_CH_2_CH_2_**OH** | **Functionality** *Alcohol* | **Fluoro-telomer Alcohol (FTOH)** |
| **F(CF_2_)_8_CH_2_CH_2_OH** | *Eight fluorinated carbons, two non-fluorinated carbons, therefore 8:2* | **8:2 Fluoro-telomer Alcohol (8:2 FTOH)** |

## Example #2 Perfluorobutane sulfonamide

| **Substance Chemical Structure** | **Question** | **Conclusion** |
| --- | --- | --- |
| **F(CF_2_)_4_SO_2_NH_2_** | **Polymer or Non- Polymer?** | **Non-Polymer** |
| **F(CF_2_)_4_SO_2_NH_2_** | **Perfluorinated?**  *Yes. All hydrogens on all four carbons are replaced with fluorine. Perfluorobutyl* | **Perfluorinated** |
| **F(CF_2_)_4_SO_2_NH_2_** | **Perfluoroalkyl Acid?**  *No. Has no acid functionality* |  |
| **F(CF_2_)_4_SO_2_NH_2_** | **Process Origin?**  *Perfluoroalkyl chain with a sulfone, -SO_2_-, spacer. Electrochemical fluorination (ECF) origin* | **ECF origin** |
| **F(CF_2_)_4_SO_2_NH_2_** | **Functionality** *Sulfonamide* | **Perfluorobutane-sulfonamide (FBSA)** |

## Example #3 Perfluorohexyl phosphonic acid

| **Substance Chemical Structure** | **Question** | **Conclusion** |
| --- | --- | --- |
| **F(CF_2_)_6_P(=O)(OH)_2_** | **Polymer or Non- Polymer?** | **Non-Polymer** |
| **F(CF_2_)_6_P(=O)(OH)_2_** | **Perfluorinated?**  *Yes. All hydrogens on all six carbons are replaced with fluorine. Perfluorohexyl* | **Perfluorinated** |
| **F(CF_2_)_6_P(=O)(OH)_2_** | **Perfluoroalkyl Acid?**  *Yes. Phosphonic, ‑P(=O)(OH)_2_, acid* | **Perfluoroalkyl acid** |
| **F(CF_2_)_6_P(=O)(OH)_2_** |  | **Perfluorohexyl phosphonic acid (C6-PFPA)** |

## Example #4 Side-chain Fluorinated Acrylate Polymer (Fluorotelomer origin)

| **Substance Chemical Structure** | **Question** | **Conclusion** |
| --- | --- | --- |
|  | **Polymer or Non-Polymer?** | **Polymer** |
|  | **Fluoropolymer?**  *No. The polymer backbone contains no fluorine bound to carbon.* |  |
|  | **Side-chain fluorinated?**  *Yes* | **Side-chain fluorinated polymer** |
|  | **Polymer Type?**  *Acrylate* | **Side-chain fluorinated acrylate polymer** |
